# Exposure to autoimmune disorders is associated with increased Alzheimer’s disease risk in a multi-site electronic health record analysis

**DOI:** 10.1016/j.xcrm.2025.101980

**Published:** 2025-02-24

**Authors:** Grace D. Ramey, Alice Tang, Thanaphong Phongpreecha, Monica M. Yang, Sarah R. Woldemariam, Tomiko T. Oskotsky, Thomas J. Montine, Isabel Allen, Zachary A. Miller, Nima Aghaeepour, John A. Capra, Marina Sirota

**Affiliations:** 1 Biological and Medical Informatics PhD Program, UCSF, San Francisco, CA, USA; 2 Bakar Computational Health Sciences Institute, UCSF, San Francisco, CA, USA; 3 School of Medicine, UCSF, San Francisco, CA, USA; 4 Department of Pathology, Stanford University, Palo Alto, CA, USA; 5 Department of Anesthesiology, Perioperative and Pain Medicine, Stanford University, Palo Alto, CA, USA; 6 Department of Biomedical Data Science, Stanford University, Palo Alto, CA, USA; 7 Department of Medicine, Division of Rheumatology, UCSF, San Francisco, CA, USA; 8 Department of Pediatrics, UCSF, San Francisco, CA, USA; 9 Department of Epidemiology and Biostatistics, UCSF, San Francisco, CA, USA; 10 Memory and Aging Center, UCSF, San Francisco, CA, USA; 11 Department of Pediatrics, Stanford University, Palo Alto, CA, USA

**Keywords:** autoimmunity, Alzheimer’s, bioinformatics, case-control, cohort, electronic health records, risk analysis, sex differences, statistical epidemiology

## Abstract

Autoimmunity has been proposed to increase Alzheimer’s disease (AD) risk, but evaluating the clinical connection between autoimmune disorders and AD has been difficult in diverse populations. We investigate risk relationships between 26 autoimmune disorders and AD using retrospective observational case-control and cohort study designs based on electronic health records for >300,000 individuals at the University of California, San Francisco (UCSF) and Stanford University. We discover that autoimmune disorders are associated with increased AD risk (odds ratios [ORs] 1.4–1.7) across study designs, primarily driven by endocrine, gastrointestinal, dermatologic, and musculoskeletal disorders. We also find that autoimmune disorders associate with increased AD risk in both sexes, but the AD sex disparity remains in those with autoimmune disorders: women exhibit higher AD prevalence than men. This study identifies consistent associations between autoimmune disorders and AD across study designs and two real-world clinical databases, establishing a foundation for exploring how autoimmunity may contribute to AD risk.

## Highlights

• Cross-trait study of >300K patients’ electronic health records at UCSF and Stanford
• Autoimmune conditions associate with greater Alzheimer’s risk
• Women with autoimmune disorders have higher risk than men with autoimmune disorders
• Association is robust in comorbidity-adjusted case-control and cohort study designs

## Graphical abstract

**Figure:**
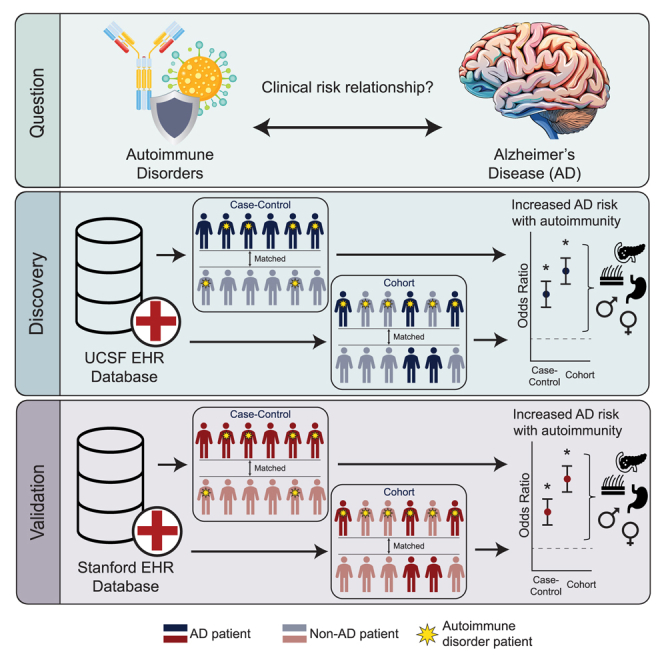

## Teaser

Ramey et al. use robust statistical approaches to identify a clinical risk association between autoimmune conditions and Alzheimer’s disease (AD). The association is consistent across UCSF and Stanford electronic health record databases and case-control and cohort study designs. The increased risk highlights immune dysregulation as a potential contributor to AD.

## Introduction

Alzheimer’s disease (AD) is a debilitating neurodegenerative disease that is accompanied by enormous social and economic burdens, and its prevalence is increasing due to the growing aging population worldwide.^1^^,^^2^ AD is characterized biologically by amyloid plaques and tau deposition in the brain, while clinical syndromic diagnoses, such as specific forms of progressive memory loss, have evolved with the development of better AD diagnostic tests and biomarkers.^3^^,^^4^ Treatments that slow cognitive decline have been a large focus of AD research over the past several years,^5^ and understanding of underlying risks and pathogenesis in order to treat the disease at an earlier stage is especially important considering that current treatments are still unable to fully rescue normal cognition.^6^

Many prior molecular and genetic studies suggest a potential role of the immune system and chronic inflammation in AD pathogenesis.^7^^,^^8^^,^^9^ Indeed, over half of the genetic variants associated with AD to date are primarily expressed in immune cells.^10^ Furthermore, several studies point to immune pathways like the NLRP3 inflammasome^11^ and complement system^12^^,^^13^^,^^14^ becoming dysregulated in AD experimental animal and human models. However, the extent of contribution of immune system dysfunction to AD remains poorly understood at the clinical phenotype level in diverse human populations. Autoimmune disorders are one potential source of chronic immune dysregulation, and their clinical risk relationship with neurodegenerative diseases like AD has yet to be fully characterized. Furthermore, autoimmune disorders exhibit a similar sex disparity to AD,^15^^,^^16^ affecting women more so than men,^17^^,^^18^^,^^19^ suggesting a potential relationship between biological mechanisms and clinical manifestations that has yet to be quantified. Therefore, studying risk relationships in individuals with autoimmune disorders and AD will provide a powerful way to understand the role of autoimmunity as a risk factor for AD overall and across sexes.

With advances in curation of real-world datasets^20^ such as electronic health records (EHRs), there is a great opportunity to investigate clinical risk relationships between many autoimmune disorders and AD. The large sample sizes that EHRs provide allow for robust analyses that can be stratified in a sex- and disease-specific manner and validated across hospital sites. Here, we quantify associations between AD and 26 autoimmune disorders in the University of California, San Francisco (UCSF) EHR system to explore the biological effects of immune dysfunction on AD pathogenesis at the phenotypic level. We show that there is a clear and consistent association between autoimmune disorders and AD risk overall and in both men and women, but that women with autoimmune disorders continue to demonstrate the highest AD prevalence. We show evidence for an increased AD risk association for specific autoimmune disorders and disorder subtypes, and we further investigate the timing of AD onset in patients with autoimmune disorders. Finally, we provide validation of the risk associations in the Stanford EHR system and control for comorbidities to demonstrate stability of the results across different study designs, medical centers, and potential confounding factors.

## Results

**Figure 1:**
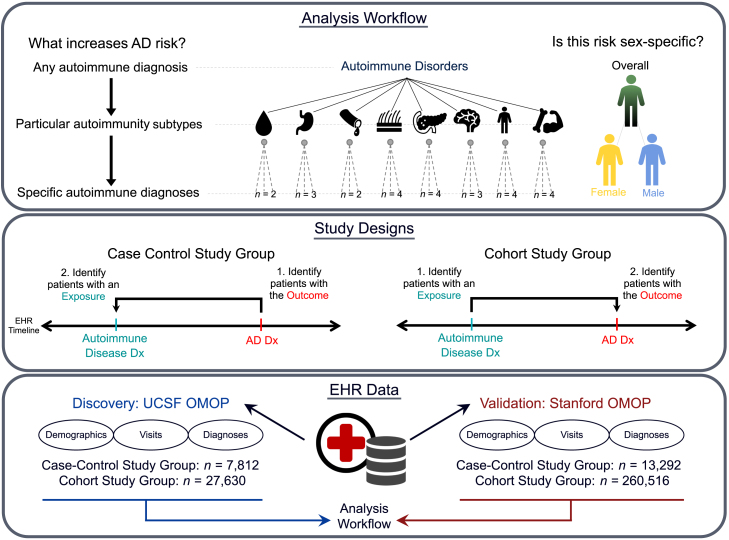
Risk analysis workflow and observational study designs We calculated AD risk in patients with autoimmune disorders using a top-down hierarchical approach in two large electronic health record (EHR) datasets. We assessed if any autoimmune disorder diagnosis, a particular autoimmune subtype diagnosis, or a specific autoimmune disorder diagnosis associated with increased AD risk. We used both case-control and cohort study designs to ensure robustness and reduce biases. For the case-control study design, we first identified patients with the *outcome* of interest (an AD diagnosis, red) and then determined which of the patients with AD also had an autoimmune diagnosis (blue). We matched the patients with AD to non-AD controls using propensity score matching on demographic, healthcare utilization, and comorbidity variables for cases and controls. For the cohort study design, we identified patients with the *exposure* first (an autoimmunity diagnosis, blue) and determined which of the exposed patients also had an AD diagnosis (red). Propensity score matching was used to match autoimmune cases to non-autoimmune controls. We used these study designs to analyze data from both the UCSF (discovery) and Stanford (validation) EHR databases. Dx, diagnosis.

We selected patients with autoimmune disorders and/or AD for case-control and cohort study designs from the UCSF and Stanford EHR databases, which contain information on over 5 million and 3.8 million patients, respectively. We identified individuals with AD and each of 26 different autoimmune disorders of interest (Table S1) using a multi-step algorithm based on standardized billing concepts confirmed by clinician review (STAR Methods). Type 1 diabetes, rheumatoid arthritis, autoimmune thyroiditis, and inflammatory bowel disease were among the most prevalent conditions in the study groups. The case-control study groups consisted of 7,812 individuals (3,906 patients with AD and 3,906 non-AD controls, Figures 1 and S1) from UCSF and 13,292 individuals (6,646 patients with AD and 6,646 non-AD controls, Figures 1 and S1) from Stanford. The cohort study groups consisted of 27,630 individuals (13,815 patients with autoimmune disorders and 13,815 non-autoimmune controls, Figures 1 and S1) from UCSF and 260,516 individuals (130,258 patients with autoimmune disorders and 130,258 non-autoimmune controls, Figures 1 and S1) from Stanford. Women made up a majority of each of our study groups, representing 61.9% (case-control) and 57.9% (cohort) of individuals in the UCSF study groups and 62.3% (case-control) and 64.9% (cohort) of individuals in the Stanford study groups. Further demographic information including lifespan and self-reported race and ethnicity values across study designs and EHR datasets is listed in Table 1.

**Table 1:** UCSF and Stanford study group demographics

UCSF					Stanford				
Case-control study design					Case-control study design				
	Non-AD controls	Patients with AD	*p* value	SMD		Non-AD controls	Patients with AD	*p* value	SMD
*n*	3,906	3,906	–	–	*n*	6,646	6,646	–	–
Sex (%)	–	–	1	<0.001	Sex (%)	–	–	1	<0.001
Female	2,416 (61.9)	2,416 (61.9)	–	–	Female	4,143 (62.3)	4,143 (62.3)	–	–
Male	1,490 (38.1)	1,490 (38.1)	–	–	Male	2,503 (37.7)	2,503 (37.7)	–	–
Unknown	0 (0.0)	0 (0.0)	–	–	Unknown	0 (0.0)	0 (0.0)	–	–
Self-reported race (%)	–	–	1	<0.001	Self-reported race (%)	–	–	1	<0.001
Asian	531 (13.6)	531 (13.6)	–	–	Asian	952 (14.3)	952 (14.3)	–	–
Black or African American	232 (5.9)	232 (5.9)	–	–	Black or African American	315 (4.7)	315 (4.7)	–	–
Native American or Alaska Native	4 (0.1)	4 (0.1)	–	–	Native American or Alaska Native	9 (0.1)	9 (0.1)	–	–
Native Hawaiian or other Pacific Islander	174 (4.5)	174 (4.5)	–	–	Native Hawaiian or Other Pacific Islander	39 (0.6)	39 (0.6)	–	–
White	2,634 (67.4)	2,634 (67.4)	–	–	White	4,182 (62.9)	4,182 (62.9)	–	–
Other	331 (8.5)	331 (8.5)	–	–	Other	813 (12.2)	813 (12.2)	–	–
Unknown/decline to state	0 (0.0)	0 (0.0)	–	–	Unknown/decline to state	336 (5.1)	336 (5.1)	–	–
Self-reported ethnicity (%)	–	–	1	<0.001	Self-reported ethnicity (%)	–	–	1	<0.001
Not Hispanic or Latino	3,672 (94.0)	3,672 (94.0)	–	–	Not Hispanic or Latino	5,732 (86.2)	5,732 (86.2)	–	–
Hispanic or Latino	234 (6.0)	234 (6.0)	–	–	Hispanic or Latino	503 (7.6)	503 (7.6)	–	–
Unknown/decline to state	0 (0.0)	0 (0.0)	–	–	Unknown/decline to state	411 (6.2)	411 (6.2)	–	–
Birth year (mean [SD])	1,935.52 (5.93)	1,935.52 (5.92)	1	<0.001	Birth year (mean [SD])	1,934.53 (10.11)	1,934.50 (10.07)	0.827	0.004
Lifespan, years (mean [SD])	80.05 (6.74)	80.07 (6.73)	0.945	0.002	Lifespan, years (mean [SD])	NA^a^	NA^a^	–	–
Autoimmune disorder status = True (*n*)	235 (6.0)	377 (9.7)	<0.001	0.136	Autoimmune disorder status = True (*n*)	415 (6.2)	575 (8.7)	<0.001	0.092

Cohort study design					Cohort study design				
	Non-autoimmune controls	Patients with autoimmune disorder	*p* value	SMD		Non-autoimmune controls	Patients with autoimmune disorder	*p* value	SMD
*n*	13,815	13,815	–	–	*n*	130,258	130,258	–	–
Sex (%)	–	–	1	<0.001	Sex (%)	–	–	1	<0.001
Female	7,994 (57.9)	7,994 (57.9)	–	–	Female	84,515 (64.9)	84,515 (64.9)	–	–
Male	5,821 (42.1)	5,821 (42.1)	–	–	Male	45,717 (35.1)	45,717 (35.1)	–	–
Unknown/decline to state	0 (0.0)	0 (0.0)	–	–	Unknown/decline to state	26 (0.0)	26 (0.0)	–	–
Self-reported race (%)	–	–	1	<0.001	Self-reported race (%)	–	–	1	<0.001
Asian	1,241 (9.0)	1,241 (9.0)	–	–	Asian	19,022 (14.6)	19,022 (14.6)	–	–
Black or African American	1,195 (8.7)	1,195 (8.7)	–	–	Black or African American	5,036 (3.9)	5,036 (3.9)	–	–
Native American or Alaska Native	85 (0.6)	85 (0.6)	–	–	Native American or Alaska Native	559 (0.4)	559 (0.4)	–	–
Native Hawaiian or Other Pacific Islander	541 (3.9)	541 (3.9)	–	–	Native Hawaiian or Other Pacific Islander	1,087 (0.8)	1,087 (0.8)	–	–
White	8,916 (64.5)	8,916 (64.5)	–	–	White	72,757 (55.9)	72,757 (55.9)	–	–
Other	1,837 (13.3)	1,837 (13.3)	–	–	Other	22,111 (17.0)	22,111 (17.0)	–	–
Unknown/decline to state	0 (0.0)	0 (0.0)	–	–	Unknown/decline to state	9,686 (7.4)	9,686 (7.4)	–	–
Self-reported ethnicity (%)			1	<0.001	Self-reported ethnicity (%)			1	<0.001
Not Hispanic or Latino	12,339 (89.3)	12,339 (89.3)	–	–	Not Hispanic or Latino	101,442 (77.9)	101,442 (77.9)	–	–
Hispanic or Latino	1,476 (10.7)	1,476 (10.7)	–	–	Hispanic or Latino	17,816 (13.7)	17,816 (13.7)	–	–
Unknown/decline to state	0 (0.0)	0 (0.0)	–	–	Unknown/decline to state	11,000 (8.4)	11,000 (8.4)	–	–
Birth year (mean [SD])	1,946.49 (13.43)	1,946.55 (13.51)	0.705	0.005	Birth year (mean [SD])	1,968.38 (21.83)	1,968.38 (21.83)	0.724	0.001
Lifespan, years (mean [SD])	69.25 (13.21)	69.10 (13.38)	0.327	0.012	Lifespan, years (mean [SD])	NA^a^	NA^a^	–	–
AD status = True (*n*)	195 (1.4)	379 (2.7)	<0.001	0.093	AD status = True (*n*)	354	579	<0.001	0.029

SMD, standardized mean difference; SD, standard deviation. A chi-squared test (with continuity correction) was used to obtain the *p* value for comparisons of categorical variables, and a one-way analysis of variance (ANOVA) test was used to obtain the *p* value for continuous variables.

a Reports of “NA” for lifespan indicate censored death information in the Stanford study groups.

To evaluate the accuracy of diagnoses in our study groups, we conducted a chart review on 50 randomly selected individuals from the UCSF study groups to confirm AD diagnoses (STAR Methods). Among the individuals whose notes had sufficient data to confidently determine AD status, there were only two false positives (individuals incorrectly classified as having AD when they did not) and no false negatives (individuals classified as not having AD when they did). Thus, the positive predictive value (PPV) for the approach was 0.82, the negative predictive value was 1.0, and the sensitivity was 1.0.

We compared the risk of being diagnosed with AD in patients with autoimmune disorders compared to non-autoimmune controls across case-control and cohort study designs in both the discovery (UCSF) and validation (Stanford) datasets. We then evaluated the risk of AD in the study groups in a sex-stratified manner and across multiple levels of autoimmune disorder stratifications to determine specific autoimmune drivers associating with AD risk.

### Autoimmune disorders are significantly associated with increased AD risk overall and within sex-specific groups

**Figure 2:**
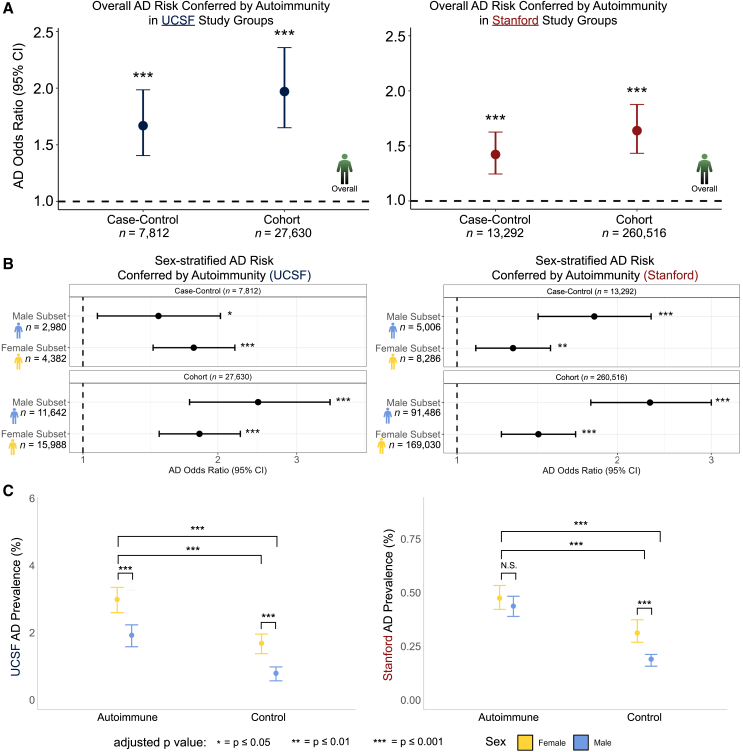
Autoimmune disorders are associated with increased AD risk across study designs and EHR datasets (A) Odds ratios quantifying AD risk in autoimmune patients versus non-autoimmune controls. We observed increased odds of AD across both the UCSF (left) and Stanford (right) datasets, and across case-control and cohort study groups within each dataset. Error bars throughout (A–C) represent 95% confidence intervals. (B) AD risk in the female- and male-only subsets of each dataset. Increased AD risk was present in both sexes in each dataset. (C) AD prevalence calculated in the cohort study designs in different sex and disorder strata. AD prevalence was higher in women with autoimmune disorders compared to many other strata groups in both the UCSF (left) and Stanford (right) data. Confidence intervals were obtained by bootstrapping.

In the case-control study groups, individuals with autoimmune disorders had significantly higher odds of an AD diagnosis compared to non-autoimmune controls in both discovery (odds ratio [OR] = 1.7, 95% confidence interval [CI] 1.4–2.0, *p* = 2.5e−9, Figure 2A) and validation (OR = 1.4, 95% CI 1.2–1.6, *p* = 1.4e−7, Figure 2A) datasets. We observed even larger AD risk associations with autoimmune disorders in the cohort study groups, where ORs were 2.0 (95% CI 1.7–2.4, *p* = 7.0e−15, Figure 2A) and 1.6 (95% CI 1.4–1.9, *p* = 1.6e−13, Figure 2A) at UCSF and Stanford, respectively. This consistent elevated risk across study designs and EHR systems suggests a consistent connection between autoimmunity and AD risk.

Since ORs and risk ratios can differ substantially in cohort studies,^21^ we additionally computed risk ratios in both the UCSF and Stanford cohort study groups. As expected, given the low prevalence of AD in each cohort, we observed similar significant effect sizes in the UCSF and Stanford cohort study groups (risk ratios ≥ 1.7, Table S2), indicating that AD risk associated with autoimmunity is not an artifact of the OR.

We next divided the discovery and validation data into female- and male-only subsets to determine if the AD risk association remained within sex-specific groups and learn if the overall risk association was primarily driven by women. In the female-only subsets of the case-control study designs, we observed significantly greater odds of an AD diagnosis in women with autoimmune disorders compared to control women at both UCSF (OR = 1.8, 95% CI 1.4–2.2, *p* = 4.1e−8, Figure 2B) and Stanford (OR = 1.3, 95% CI 1.1–1.5, *p* = 3.0e−3, Figure 2B). Similarly, among women in the cohort study groups, we observed significantly greater AD risk in women with autoimmune disorders at UCSF (OR = 1.8, 95% CI 1.5–2.2, *p* = 4.9e−9, Figure 2B) and at Stanford (OR = 1.4, 95% CI 1.2–1.7, *p* = 1.2e−5, Figure 2B). There were also strong associations between autoimmunity and AD in the male-only cohorts, where all ORs were significant across case-control and cohort study designs (OR ≥ 1.5, Figure 2B), indicating higher risk in men with autoimmune disorders compared to control men across both EHR systems. The increased AD risk association observed in both men and women with autoimmune disorders suggests that the relationship between AD and autoimmunity is not present solely in one sex.

### AD prevalence is increased in the presence of autoimmune disorders, but women remain the most affected

While autoimmunity associates with AD risk within both sexes, we next tested whether the risk association was stronger in one sex than the other. Furthermore, we wanted to determine if the presence of autoimmunity diminished the well-documented AD sex disparity wherein women develop AD more often than men. To address these questions, we conducted an AD prevalence analysis within the cohort study groups of our discovery and validation datasets. Due to the smaller number of men compared to women in our data, we conducted 1:1 matching of women to men based on demographic variables (STAR Methods) and computed AD prevalence across sex and autoimmunity stratifications. In our discovery dataset (*n* = 23,284), women with autoimmune disorders had the highest AD prevalence at 3.0%, followed by men with autoimmune disorders at 1.9%, control women at 1.7%, and finally control men at 0.79% (Figure 2C). While the absolute prevalence values were lower at Stanford, likely due to younger patients being included in the cohort study group because of censored age information (STAR Methods), there was a similar pattern in the validation dataset (*n* = 182,972). Women with autoimmune disorders had the highest AD prevalence at 0.47%, followed by men with autoimmune disorders at 0.43%, control women at 0.31%, and control men at 0.19%. As expected, the prevalence was significantly higher in control women compared to control men (UCSF corrected *p* = 6.2e−5; Stanford corrected *p* = 1.1e−3), corroborating well-documented sex disparities in AD (Figure 2C). We also found that women with autoimmune disorders exhibited a higher AD prevalence than men with autoimmune disorders in the UCSF dataset (corrected *p* = 9.9e−4). The AD prevalence difference between autoimmunity patients of different sexes was roughly equal in magnitude to the difference between control patients of different sexes (1.1% versus 0.91%), suggesting that sex disparities in AD remain present, even when autoimmunity associates with greater risk in both men and women. The intersex comparison in autoimmune patients in the Stanford dataset was not significant (*p* = 0.4), but women with autoimmune disorders did exhibit a slightly higher AD prevalence than the corresponding men, supporting the trend that women continue to bear more risk for AD even when a significant immune perturbation like autoimmunity is at play.

### Specific autoimmune disorder subtypes are associated with greater AD risk

**Figure 3:**
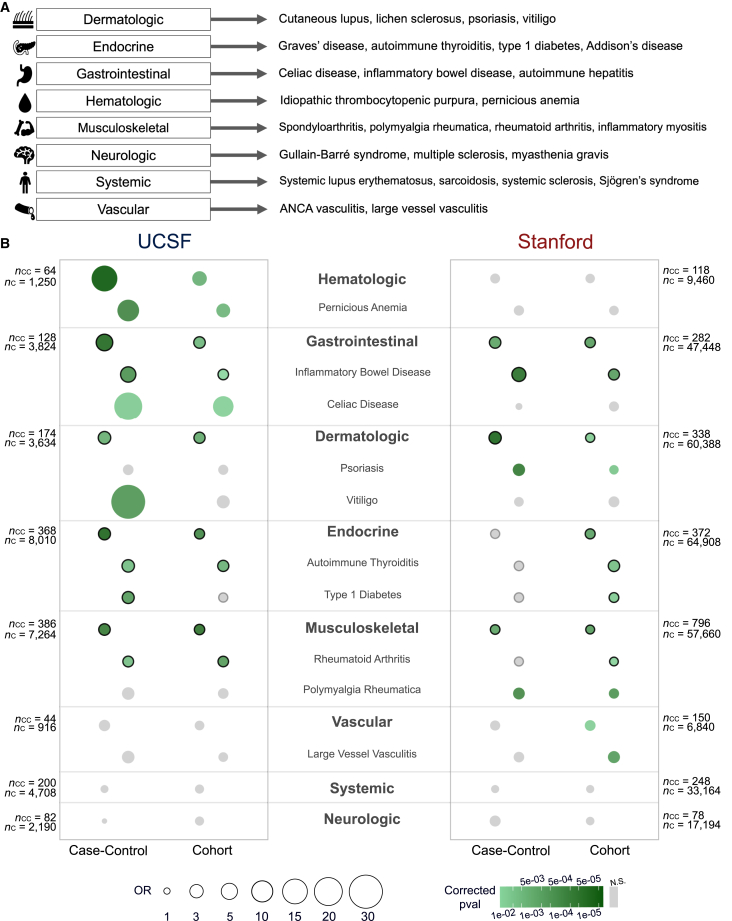
Specific autoimmune disorder subtypes and diseases associate with increased AD risk (A) Autoimmune disorders can be grouped into subtypes based on physiological symptomatology. We used eight subtypes in our analysis. (B) Odds ratios quantifying AD risk in patients with different autoimmune disorder subtypes and specific autoimmune disorders compared to controls. Disorder subtypes are in bold, and the specific disorders that fall into each subtype category are listed below. Only individual autoimmune disorders that had statistically significant associations are shown. The larger and darker the circle is, the greater the effect size and significance of the odds ratio, respectively. Circles that are outlined in black represent agreement of risk signal across study groups at more than one EHR site. N.S., not significant after multiple testing correction; CC, case-control; C, cohort.

Next, to determine if specific classes of autoimmune disorders are more strongly associated with AD risk, we divided the 26 autoimmune disorders into subtypes based on the organ system each one primarily affects. This resulted in eight disease subtype categories: musculoskeletal, gastrointestinal, dermatologic, systemic, vascular, hematologic, neurologic, and endocrine (Figure 3A). We computed the AD ORs for individuals with these disease subtypes in each of the case-control and cohort study designs across UCSF and Stanford datasets. In the overall case-control and cohort study designs of the UCSF dataset, autoimmune disorders in the gastrointestinal, hematologic, endocrine, musculoskeletal, and dermatologic categories were significantly associated with an increased risk for AD, all with ORs ≥ 1.9 (Figure 3B). Systemic, vascular, and neurologic disease subtypes did not significantly associate with increased AD risk (Figures 3B and S3), potentially because we were underpowered to detect risk associations for these subtypes (Table S3). They also potentially suggest that AD may interact with other neurological conditions or broadly acting conditions in the body like systemic and vascular autoimmune disorders to modulate risk.

Several of the autoimmune disorder groups that were significantly associated with AD risk in the UCSF dataset were validated in the Stanford dataset in one or both study designs. Gastrointestinal (case-control and cohort), endocrine (cohort), musculoskeletal (case-control and cohort), and dermatologic (case-control and cohort) disorders all associated with significantly more AD risk in autoimmune patients compared to non-autoimmune controls in the Stanford EHR system, with ORs ≥ 1.5 (Figure 3B).

Next, we investigated if specific autoimmune disorders were driving the subtype and overall risk associations we saw. Disorders that were significantly associated with increased AD risk in at least two study groups and across more than one EHR system included inflammatory bowel disease (gastrointestinal category), autoimmune thyroiditis (endocrine category), type 1 diabetes (endocrine category), and rheumatoid arthritis (musculoskeletal category). ORs for each of these disorders were ≥1.4 (Figures 3B and S3). These results on individual disorders point to specific mechanisms that may contribute to the interplay of risk between autoimmunity and AD.

### Autoimmunity remains significantly associated with increased AD risk when accounting for comorbidities, with slightly smaller effect sizes

We next evaluated whether differences in chronic comorbidities between groups influenced the association of autoimmune disorders with AD risk by matching individuals on the Charlson-Deyo Comorbidity Index.^22^^,^^23^ The comorbidities considered included type 2 diabetes, various cancers, chronic pulmonary disease, and cardiac conditions; we did not include dementia and rheumatic diseases in the index calculation, given the focus on these as exposures or outcomes of our study (STAR Methods). After controlling for these comorbidities using propensity score matching among groups, the associations between autoimmune disorders and AD were still significant. At UCSF, in the case-control study group, we observed an OR of 1.4 (95% CI 1.2–1.7, adjusted *p* = 3.7e−5, Figure S11 and Table S4), and in the cohort study group, we observed an OR of 1.3 (95% CI 1.1–1.5, adjusted *p* = 1.8e−3, Figure S11 and Table S4). At Stanford, we observed an OR of 1.2 (95% CI 1.1–1.4, adjusted *p* = 5.1e−3, Table S4) in the case-control study group and 1.3 (95% CI 1.1–1.5, adjusted *p* = 1.3e−4, Table S4) in the cohort study group. Given the consistency of risk associations across study groups and EHR sites, these results suggest that underlying chronic comorbidities are not driving the AD risk associations we observed.

While not every disorder subtype or individual disorder (e.g., type 1 diabetes and rheumatoid arthritis) remained significant across every comorbidity-adjusted analysis, many subtypes and individual disorders that were associated with increased AD risk were recapitulated in different study groups. The subtypes that remained most consistently associated with greater AD risk, particularly in the UCSF study groups, were the endocrine and dermatologic disorder subtypes (Table S4). Gastrointestinal and musculoskeletal subtypes were also significantly associated with increased AD risk in three out of the four study groups across EHR sites after comorbidity adjustment (Table S4). In terms of recapitulation of individual disorder risk signals, autoimmune thyroiditis remained significantly associated with increased AD risk particularly in the UCSF study groups after comorbidity adjustment (Table S4), while inflammatory bowel disease was associated with increased risk across three of the four study groups after adjustment (Table S4).

### AD risk from disease subtypes exhibits variable sex-specific effects

Next, we stratified the disease subtype and individual disease analyses by sex to determine if any particular risk association was driven more by one sex compared to the other. Some of these stratified analyses had low statistical power (Table S3), but many disease subtypes exhibited sex-specific risk signals. For example, the endocrine category of disorders was predominantly significant among women, exhibiting a significant AD risk effect in the female-specific UCSF study groups and female-specific Stanford cohort study group, where all ORs were ≥1.8 (Figure S2). In the dermatologic category, male-specific AD risk was higher than that of the female-specific comparison in most study groups. For example, in the UCSF cohort study group, men with dermatologic autoimmune disorders exhibited an OR of 1.5 (95% CI 1.4–9.3, corrected *p* = 0.04, Figure S2), while women exhibited an OR of 2.0 that was no longer statistically significant after multiple testing correction (95% CI 1.1–3.8, *uncorrected p* = 0.02, Figure S2). Similarly, in both Stanford study groups, men with dermatologic autoimmune disorders were at greater risk than matched controls (case-control OR = 7.8, 95% CI 3.4–18.5, corrected *p* = 4.3e−7; cohort OR = 2.6, 95% CI 1.5–4.7, corrected *p* = 1.9e−3, Figure S2) compared to women (case-control OR = 1.6, 95% CI 0.9–3.0, corrected *p* = 0.94; cohort OR = 1.2, 95% CI 0.8–1.8, corrected *p* = 3.5, Figure S2). Interestingly, in the case-control study group at UCSF, we also observed increased female-specific AD risk for individuals with dermatologic autoimmune disorders (OR = 6.4, 95% CI 2.4–18.3, corrected *p* = 4.4e−4, Figure S2), perhaps indicating variable sex-specific effects for this category of diseases.

Clear sex-specific differences in the risk associated with individual autoimmune disorders were harder to identify given a lack of statistical power due to small sample sizes after stratifying by both sex and disorder (Table S3). Nonetheless, significant female-specific AD risk associations with autoimmune thyroiditis were present in the cohort study groups of both EHR datasets (ORs ≥ 2.4, Figure S4), potentially driving the female-specific risk in the endocrine category of diseases. Several other diseases associated with increased AD risk significantly across some study groups, but not across others (Figure S4), or exhibited different directions of sex-specific effects across the study groups (Figure S4). This highlights the need for better-powered studies of sex differences in the interaction between autoimmunity and AD in the future.

### Sensitivity analyses support risk associations between autoimmune disorders and AD

We performed additional sensitivity analyses to address the potential presence of confounders and further test the robustness of the AD risk associations we identified.^24^ The sensitivity analyses were performed primarily in the discovery dataset, as we wanted to verify the risk associations in the smaller UCSF study groups before performing validations in the Stanford study groups. Our first sensitivity analysis was to apply an age cutoff in our study designs to quantify AD risk due to autoimmunity in two older sub-populations of individuals. The first cutoff only included individuals in the study groups that had reached an age of 65 years or older. When comparing autoimmune patients to non-autoimmune controls in this >65 dataset, we observed an AD OR of 1.6 (95% CI 1.4–2.0, *p* = 9.7e−9) in the case-control study group and 2.0 (95% CI 1.6–2.4, *p* = 1.7e−14) in the cohort study group (Table S4). Raising the cutoff to 80 years of age or older also resulted in a consistent AD risk association with autoimmune disorders (case-control OR = 1.7, 95% CI 1.4–2.2, *p* = 1.7e−6; cohort OR = 2.4, 95% CI 1.8–3.1, *p* = 1.8e−12, Table S4), indicating that our signal was robust to the age range of patients in our discovery dataset.

To focus our risk analysis on only late-onset AD cases, we removed matched pairs of individuals from our study groups in which one individual within the pair had an AD diagnosis prior to 65 years of age. Even after the removal of these individuals, we observed elevated AD risk in autoimmune patients compared to unexposed controls. The OR was 1.7 (95% CI 1.4–2.0, *p* = 3.9e−9) in the case-control study group and 2.0 (95% CI 1.7–2.4, *p* = 1.9e−14) in the cohort study group (Table S4), highlighting the elevated late-onset AD occurrence in those with autoimmunity.

Next, we matched individuals based on their total number of comorbidities (STAR Methods), and we continued to observe significant AD risk associations in autoimmune patients compared to non-exposed controls across study groups. We detected an OR of 1.3 (95% CI 1.1–1.5, adjusted *p* = 2.1e−2, Figure S11 and Table S4) in the case-control study group and 1.3 (95% CI 1.1–1.5, *p* = 1.3e−3, Figure S11 and Table S4) in the cohort study group, indicating that the increased AD risk association was robust to cumulative comorbidity burden.

We next performed a sensitivity analysis to address possible confounder and collider effects that healthcare utilization can cause in EHR systems, as these effects can exaggerate or attenuate differences between exposure and outcome groups. Healthcare utilization has also been associated with measures of different social determinants of health.^25^ In addition to matching on demographic variables (STAR Methods), we matched individuals based on the similarity of the length of time between their first and last hospital visit date (Figures S9 and S10). After conducting this matching and recomputing ORs in each study group, autoimmune disorders were still significantly associated with an increased risk for AD (case-control OR = 1.3, 95% CI 1.1–1.5, *p* = 6.0e−3; cohort OR = 1.4, 95% CI 1.2–1.7, *p* = 5.2e−6, Table S4). Matching on each patient’s frequency of visits per year also resulted in consistent increased risk associations between autoimmune disorders and AD (case-control OR = 1.3, 95% CI 1.1–1.6, *p* = 3.7e−4; cohort OR = 1.5, 95% CI 1.3–1.7, *p* = 2.0e−6, Table S4), highlighting the connection between autoimmunity and AD at the clinical level even when adjusting for different healthcare utilization measures.

In the next sensitivity analysis, we recomputed ORs in each study group without including age at death as a variable in matching to determine the influence of lifespan on risk, as differences in lifespan can lead to selection bias (study group matching and quality control in STAR Methods). This resulted in an AD OR of 1.3 (95% CI 1.1–1.5, *p* = 7.1e−4, Table S4) in the case-control study group and 2.1 (95% CI 1.8–2.5, *p* = 4.7e−17, Table S4) in the cohort study group, indicating that the risk association was robust to leaving total lifespan information out of the matching criteria.

Finally, reverse causality can occur when the outcome precedes the exposure but is not recorded properly in the EHR. Reverse causality has presented a challenge in some previous EHR studies of dementia.^26^ To evaluate the potential for reverse causality in our study, we removed individuals from the study groups who were diagnosed with AD within one year of being diagnosed with an autoimmune disorder in addition to removing these individuals’ matched pairs. This was to account for potential inaccuracies in diagnosis timing and the assessment of onset. Under the 1-year filtering criterion, the OR in the case-control study group was 1.3 (95% CI 1.1–1.6, *p* = 4.9e−3, Table S4), and the OR in the cohort study group was 1.5 (95% CI 1.3–1.9, *p* = 3.3e−6, Table S4), suggesting significantly elevated AD risk after an autoimmune diagnosis in patients. We also evaluated results under a more extreme filter, where we removed all AD cases within 3 years of an autoimmune diagnosis, in addition to these individuals’ matched controls. Again, we observed ORs greater than one. The OR in the cohort study group was 1.3 (95% CI 1.1–1.6, *p* = 7.4e−3, Table S4), and the OR in the case-control study group was 1.1 (95% CI 0.9–1.3, *p* = 0.4, Table S4). This stricter 3-year filtering criterion significantly reduced our power to detect an association in the case-control study group (power = 0.13; Table S4), and the 3-year time interval likely resulted in the removal of more individuals who developed AD well after an autoimmunity diagnosis. These results highlight that reverse causality is likely not the driver of the associations.

Several disease subtypes and individual disease associations seen in the main UCSF analysis were replicated in many of these sensitivity conditions, including the increased AD risk due to endocrine, gastrointestinal, and musculoskeletal disorders driven by type 1 diabetes, autoimmune thyroiditis, inflammatory bowel disease, and rheumatoid arthritis, and these are further highlighted in Table S4.

### Sex is associated with accelerated AD onset over time

**Figure 4:**
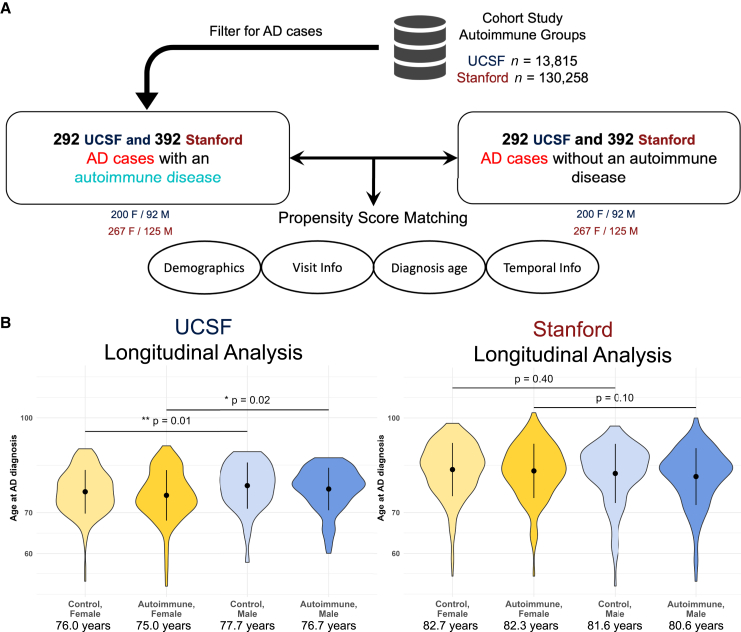
AD onset is earlier in females, with potential acceleration from autoimmune disorders (A) Study design for the longitudinal AD onset analysis, conducted using data from the UCSF and Stanford EHR datasets. M, male; F, female. (B) Distributions of AD diagnosis age among individuals with and without autoimmune diseases in the longitudinal cohorts, stratified by sex. The dot indicates the mean, and the black lines indicate the standard deviation. The mean age at diagnosis is reported under each group. Within the autoimmune and control subgroups, females were diagnosed with AD at a younger age than males. The age of AD onset for each group of individuals with autoimmune diseases was earlier than the controls, but the statistical significance of these differences was only moderate due to the small sample size.

In addition to examining the presence or absence of AD in patients with autoimmune disease through risk analyses, we also tested whether autoimmune disorders were associated with the timing of AD onset. We hypothesized that having an autoimmune disorder would be associated with a decrease in the age at which people were diagnosed with AD, potentially due to the early presence of chronic inflammation that autoimmune disorders may cause. We constructed new longitudinal cohorts for this analysis consisting only of patients with AD with and without autoimmune disorders (STAR Methods). This resulted in 292 autoimmune patients matched to 292 controls at UCSF (*n* = 584 total patients with AD, Figure 4A) and 392 autoimmune patients matched with 392 controls at Stanford (*n* = 784 total patients with AD, Figure 4A). We first compared the distributions of AD diagnosis age among patients. In the UCSF longitudinal cohort, the average age at which autoimmune patients were diagnosed with AD was 75.6 years, compared to the controls at 76.5 years (Figure S5A). In the Stanford longitudinal cohort, the average AD diagnosis age was 81.8 years in autoimmune patients compared to 82.4 years in controls (Figure S5A). While the AD diagnosis age was lower in autoimmune patients in each dataset, the statistical significance of the differences between age distributions was only moderate (UCSF *p* = 0.11, Stanford *p* = 0.17, Figure S5A), likely because of small sample sizes (Table S3). The 0.6- to 1-year difference in diagnosis age we observed is nonetheless striking, given the often rapid symptomatic decline^27^ of individuals with AD. Even being diagnosed with AD half a year earlier could be extremely impactful for patients and their quality of life, and we expect the difference in diagnosis age would be statistically significant in larger longitudinal cohorts.

We also observed differences in AD onset age when we stratified individuals by both sex and autoimmune disorder. Starting in the UCSF longitudinal cohort, women with autoimmune disorders exhibited significantly younger AD onset ages (mean 75.0 years, Figure 4B) compared to men with autoimmune disorders (mean 76.7 years, *p* = 0.017, Figure 4B). Similarly, control women exhibited significantly younger AD onset ages (mean 76.0 years, Figure 4B) compared to control men (mean 77.7 years, *p* = 0.011, Figure 4B). Comparing autoimmune patients to matched controls within each sex revealed younger onset ages for individuals with autoimmune disease, but again these were not statistically significant given the small sample size (female *p* = 0.19, male *p* = 0.28, Figure 4B). Results from the Stanford longitudinal cohort all agreed directionally with the UCSF results (Figure 4B).

Finally, we performed a survival analysis to determine if the risk of developing AD in different stratifications varied over time. We used a Cox proportional hazard model for the analysis with sex and autoimmunity presence as covariates. Having an autoimmune disorder resulted in hazard ratios (HRs) greater than 1.0 for individuals developing AD over time (UCSF HR = 1.1, 95% CI 1.0–1.3, *p* = 0.16; Stanford HR = 1.0, 95% CI 0.9–1.2, *p* = 0.5, Figure S5B), but again, likely due to low statistical power, these differences did not reach statistical significance. However, given the consistent directional association between autoimmune disorders and AD risk, better-powered analyses of this relationship are warranted. Power statistics for all analyses in the current study are reported in Table S3, highlighting places where better-powered studies in other clinical databases are needed.

## Discussion

We have shown that autoimmune disorders are associated with an increased risk of being diagnosed with AD in case-control and cohort study designs across two different EHR systems, suggesting that autoimmunity is a risk factor for AD. We observed a consistently increased risk signal in our study groups, both overall and in female- and male-specific subsets, suggesting that autoimmunity is associated with increased AD risk regardless of sex. Interestingly, in our AD prevalence analysis, females with autoimmune disorders exhibited increased AD prevalence compared to males with autoimmune disorders, suggesting that both autoimmunity and sex are connected to AD risk. When adjusting for multiple sensitivity conditions including comorbidities, reverse causality, and healthcare utilization disparities, the overall association of autoimmune disorders with increased AD risk was robust across study groups. There were some differences in effect sizes in the different sensitivity analyses; however, even the lowest significant effect size we observed suggests a clinically meaningful increase in AD risk for those affected by autoimmune conditions. Additionally, given the nature of unpredictable data capture in EHRs, adjusting for certain conditions can result in under-estimations of risk. We therefore find it encouraging that we observed increased AD risk in individuals with autoimmune conditions regardless of whether we adjusted for an array of sensitivity conditions or left our estimates unadjusted. In aggregate, these results provide strong evidence for the association of autoimmune disorders with increased AD risk. Furthermore, we found preliminary evidence that sex and autoimmunity not only influence the risk of AD but also the timing of AD onset. Larger longitudinal studies are needed to provide insight into these temporal dynamics.

We observed specific subtypes of autoimmune disorders that are associated with AD in patients. While it is impossible to infer causality from an associative study, these classes of disorders suggest the potential presence of shared pathophysiology between autoimmunity subtypes and AD. For example, metabolic dysfunction has been highlighted in previous AD pathogenesis studies,^28^ so it is possible that metabolic pathways exist that link endocrine autoimmune disorders, which can strongly affect metabolism, and AD pathogenesis. Similarly, there have been documented links between changes in the gut-brain axis^29^ and microbiome^30^ that are associated with AD in previous work, which could relate AD pathogenesis to biological pathways involved in gastrointestinal autoimmune disorders. Depending on the autoimmune disorder subtype and individual autoimmune disorders we analyzed, we also observed some sex-specific risk associations, highlighting that autoimmune disorders that affect certain physiological systems may manifest differently in females compared to males. Further studying these relationships may provide insight into the mechanisms that cause or exacerbate AD in a sex-specific manner.

Our findings validate and provide new insights on previous work. Recently, Miller et al.^31^ found increased prevalence of inflammatory bowel disease in a small cohort of late-onset AD cases; our results support this pattern in a much larger clinical dataset. Furthermore, a study of the Swedish National Patient Register^32^ highlighted increased incidence rates of several autoimmune disorders including hypothyroidism/thyroiditis, type 1 diabetes, Addison’s disease, Sjӧgren’s syndrome, and pernicious anemia in patients with dementia. We were able to corroborate several of these signals with our analysis; we also found type 1 diabetes and a form of thyroiditis as significantly associated with risk. We further built upon these findings by analyzing disease subtypes to identify physiological systems that may be involved in AD pathogenesis, and we conducted extensive age of onset and sex-specific analyses to characterize AD risk in individuals with autoimmune disorders. Finally, a recent study^33^ indicated increased AD hazard linked to four major autoimmune disorders (rheumatoid arthritis, multiple sclerosis, psoriasis, and inflammatory bowel disease) in a longitudinal UK Biobank cohort. Our results expand on this study by investigating additional autoimmune disorders and sex as a biological variable. Compared to the individuals in the UK Biobank and Swedish studies, our cohort of patients is also more diverse, suggesting generalizability of the increased risk association between autoimmune disorders and AD across diverse humans.

Other recent studies have evaluated the relationship between immune system activity and forms of dementia. A large nation-wide Danish cohort study found a significant risk association between autoimmune disorders and AD, although with a small effect size after infections were accounted for in patients.^34^ Infections and autoimmune disorders are widely thought to be linked, most notably in conditions like Epstein-Barr virus and multiple sclerosis.^35^ This makes it difficult to fully disentangle their interactions and how they relate to risk of various forms of dementia, even in targeted studies. It is possible that infections and autoimmunity both contribute to dementia risk separately as they push the immune system to the extremes—infections weaken the immune system, while autoimmune disorders cause immune system hyperactivity. This “Goldilocks” hypothesis—where an imbalance of the immune system in one direction or the other may prime the body to develop AD or other dementias—has been supported by mechanistic studies highlighting that both under- and overexpression of certain inflammatory markers may contribute to AD pathology.^36^^,^^37^

Previous studies, like those in the Danish cohort^34^ and others,^38^ that have reported associations between autoimmune disorders and AD with small effect sizes have also typically not incorporated relevant variables into sensitivity analyses. For example, healthcare utilization can act as a colliding variable. We have shown that our results are robust when accounting for multiple measures of healthcare utilization such as the length of EHR record and doctor visit frequency. These prior studies were also conducted in predominantly Western European cohorts, whereas we have shown in more diverse case-control and cohort study groups that there is a strong association between autoimmune conditions and AD. Given differences in immune system genetic variation and immune system activity, the risk of AD associated with autoimmunity could vary between individuals with different genetic ancestries, but this warrants more research.

Prior work^26^ has also pointed out the potential for reverse causality in relationships between autoimmunity and AD. To evaluate this, we ran a sensitivity analysis to test the potential contribution of reverse causality to risk associations in our study. We continued to observe a strong association even after removing patients who were diagnosed with an autoimmune condition and AD within a close time interval. Effect sizes were slightly decreased in our reverse causality analysis likely due to the removal of patients who were indeed diagnosed with AD separately from and well after an autoimmunity diagnosis, but ORs did not fall below 1.3 when we were powered to detect an association. These increased risk associations were prominent across multiple study groups and filtering criteria, leading us to conclude that there is a risk association specific to autoimmune disorders and AD later in the lifespan. It is also possible that risk for AD after an autoimmune disorder diagnosis varies across time, and it could potentially decrease after the initial elevation due to AD resilience or other factors.^39^ This likely varies across conditions and also warrants further study.

By using two different study designs (case-control and cohort) in both a discovery and independent validation dataset, we were able to combat common problems of selection bias,^40^ data inaccuracies,^41^ and confounding that can be present in EHR studies, and this further supports the robustness of the associations we discovered. We also found significant risk ratios in our cohort study groups in addition to ORs. Our workflow, which we make publicly available, can be further applied not only to autoimmune disorders and their risk associations with other neurological diseases but also to any two disease types of interest that can be captured in an EHR data system.

### Limitations of the study

The current study has several limitations that should be considered when evaluating our results. First, the groupings used for the disease subtype analysis are imperfect. Since the exact underlying mechanism of many of the autoimmune disorders we investigated remains unknown, grouping by physiological system enabled us to study these diseases in aggregate, but it reflects only one dimension along which each autoimmune disorder is related to the others. Other dimensions of interest could include peripherally versus centrally acting conditions or conditions that are known to affect the same immune cell type. Another caveat is the presence of censored death information in the validation dataset. This may have resulted in the incorporation of younger patients in the Stanford study groups who had yet to develop either AD or a later-onset autoimmune disorder, and as such, this may have deflated the ORs in the Stanford study groups. It is possible that this led to less agreement in risk associations between UCSF and Stanford particularly for the subtype and individual disorder analyses. Furthermore, while we matched individuals based on lifespan and/or birth year within each of our study groups, future work could include age-matching between discovery and validation datasets to enable even more direct comparisons.

Our analysis also did not include stratifications based on treatments. We anticipate that these treatments could potentially counteract elevated AD risk in individuals with autoimmune disorders, and they require further study. Indeed, several classes of drugs that can be used to treat autoimmune disorders such as corticosteroids^42^ and tumor necrosis factor inhibitors^43^^,^^44^ are suggested to decrease dementia risk and severity. We also note that using billing codes for the identification of diagnoses can be prone to misclassification errors. We have mitigated this by using multiple study designs and two different EHR databases for validation. We also carried out a chart review for AD diagnoses and report strong PPV and other performance statistics that support sufficient accuracy of the phenotyping for our analyses. The large number of autoimmune conditions considered made a chart review for autoimmune disorders out of scope for the current work. Given the strong performance of the AD algorithm and previous work phenotyping autoimmune diseases from EHRs,^45^^,^^46^ we were confident that, while there may be some misclassification, our phenotyping algorithm was sufficiently accurate for our study. We also note that we were limited to the information captured within the EHR and that any conditions not captured there were not included in the analysis.

Finally, we note that the stratified analyses we conducted were less likely to yield consistent significant results across sites and study designs. Much of this was the result of reduced power due to the small sample sizes in the stratified cohorts. As expected, this also occasionally yielded very high ORs and large CIs. Nonetheless, the results were consistent in their direction, and many of these analyses highlight promising specific hypotheses for further validation and molecular characterization.

In summary, autoimmune disorders have a consistent association with AD in both men and women in two large real-world EHR datasets. This study illustrates the usefulness of EHRs for cross-trait analyses, and it also informs further mechanistic hypotheses about the molecular processes that may go awry in the interaction of the immune and nervous systems to promote AD pathogenesis. Further risk factor analyses for debilitating neurological conditions such as AD will empower clinicians to inform patients of their risk profiles, and, ultimately, deeper understanding of these connections between risk and disease can also empower patients themselves to make lifestyle changes or take relevant treatments that can help avoid or delay disease.

Our results highlight several future directions for further understanding of the risk relationship between autoimmune disorders and AD. First, quantifying how AD risk varies based on differing levels of autoimmune disorder severity and duration is needed. We hypothesize that more severe forms of autoimmune disorders may confer the most AD risk, and perhaps treatments to alleviate more severe disorders may decrease risk. Additionally, considering the chronic nature and onset age of many autoimmune disorders may shed more light on the temporal dynamics of the two traits interacting throughout the lifespan. Finally, integrating clinical risk analyses with other data modalities, such as genetics and proteomics, will provide more molecular insight into the link between AD and autoimmune disorders and help to fully elucidate the basis for the consistent risk seen at the phenotypic level in human populations.

## Resource availability

### Lead contact

Further information and requests for resources and reagents should be directed to and will be fulfilled by the lead contact, Marina Sirota (Marina.Sirota@ucsf.edu).

### Materials availability

This study did not generate new unique reagents.

### Data and code availability

The de-identified patient data reported on in this study cannot be deposited in a public repository because of patient data sharing privacy and ethical limitations. Please contact the UCSF Clinical Data resources team and the Stanford STARR-OMOP team for more information about access. All analysis of University of California, San Francisco and Stanford University EHR data was performed under the approval of the Institutional Review Boards from University of California, San Francisco and Stanford University, respectively. All clinical data were de-identified, and written informed consent was waived by the institutions. Medical billing concept codes used to identify patients in external EHR databases can be found in Tables S6 and S7. All patient and demographic data curation from the UCSF and Stanford EHR systems was performed using Microsoft SQL server and the DBI (v1.1.3) and odbc (v1.3.4) packages in R. Discovery data were last curated from the UCSF OMOP database on August 4th, 2023, and validation data were last curated from the Stanford OMOP database on December 12th, 2023. Data representing processed patient information that can be visualized in the figures can be found in Table S8.

Data cleaning, matching, and analysis steps were conducted using R version 4.1.3, and plots were created with the ggplot2 package (v3.4.2). All code not limited by patient data sharing permissions and proprietary clinical data structures can be found at https://github.com/gramey02/AD_AID_Project. Any additional information required to reanalyze the data reported in this paper is available from the lead contact upon request.

## Acknowledgments

We would like to thank members of J.A.C.’s, M.S.’s, and N.A.’s labs for their feedback throughout this work. We acknowledge our funding sources, including 10.13039/100000049NIA R01AG060393, 10.13039/100000069NIAMS P30 AR070155, F30 Fellowship 1F30AG079504-01, and the UCSF Discovery Fellows. We would also like to acknowledge the National Institute On Aging of the 10.13039/100000002National Institutes of Health award number 1F31AG090013-01 and the use of the UCSF Information Commons and UCSF Research Analysis Environment computational research platforms. Through these platforms, the project was supported by the 10.13039/100006108National Center for Advancing Translational Sciences, National Institutes of Health, through UCSF-CTSI grant number UL1 TR001872. Its contents are solely the responsibility of the authors and do not necessarily represent the official views of the NIH.

## Author contributions

A.T., Z.A.M., M.S., and G.D.R. conceptualized this research project. G.D.R. investigated and curated all UCSF data and performed all UCSF statistical and computational analysis. T.P. performed all Stanford validation steps, including data curation and statistical/computational analysis. M.M.Y. provided valuable clinical input on EHR methodology and clinical billing codes. G.D.R. and A.T. wrote the manuscript, and G.D.R. created visualizations. J.A.C. and M.S. provided mentorship, review/editing of writing, and funding for the work. I.A., N.A., T.J.M., T.T.O., and S.R.W. provided expertise and manuscript feedback.

## Declaration of interests

The authors declare no competing interests.

## STAR★Methods

### Key resources table

**Table:** 

REAGENT or RESOURCE	SOURCE	IDENTIFIER
**Deposited data**		

Original Code	This paper	https://github.com/gramey02/AD_AID_Project
Supplementary Figures and Tables	This paper	https://github.com/gramey02/AD_AID_Project

**Software and algorithms**		

R version 4.1.3	R Development Core Team	https://www.r-project.org/
MatchIt (v4.5.3)	Comprehensive R Archive Network	https://cran.r-project.org/package=MatchIt
epitools (v0.5–10.1)	Comprehensive R Archive Network	https://cran.r-project.org/package=epitools
survival (v3.5-5)	Comprehensive R Archive Network	https://cran.r-project.org/package=survival
survminer (v0.4.9)	Comprehensive R Archive Network	https://cran.r-project.org/package=survminer
ggplot2 (v3.4.2)	Comprehensive R Archive Network	https://cran.r-project.org/package=ggplot2
DBI (v1.1.3)	Comprehensive R Archive Network	https://cran.r-project.org/package=DBI
odbc (v1.3.4)	Comprehensive R Archive Network	https://cran.r-project.org/package=odbc
tableone (v0.13.2)	Comprehensive R Archive Network	https://cran.r-project.org/package=tableone

### Experimental model and study participant details

#### Data collection

We identified 26 autoimmune disorders of interest (Table S1) for our study based on prior literature^31^ and prevalence in the general population. Individuals with each autoimmune disorder were identified by multi-step procedure based on standardized billing concepts confirmed by clinician review. In the first step, string-matching was used to link autoimmune disorder names (Table S5) with billing concepts. This approach has been used in other studies to begin relevant billing concept selection.^47^^,^^48^ The initial string search aided in eliminating billing concepts that were too general or irrelevant for a particular condition of interest. Concepts were subsequently standardized for use in the UCSF EHR database^49^ which is based on the Observational Medical Outcomes Partnership (OMOP) Common Data Model (CDM) and primarily uses standardized Systemized Nomenclature of Medicine (SNOMED) concept encodings. All billing concepts were examined by UCSF rheumatologists to confirm validity and relevance of each concept to each autoimmune disorder of interest. We compiled a final list of 878 autoimmune billing concepts that we make available to readers for ease of reproducibility (Table S6). We identified patients who had these concepts present in their UCSF medical record to construct our discovery dataset, and we used identical concepts to identify patients from the Stanford OMOP EHR database^50^ for our validation dataset. We identified patients with AD in a similar manner to autoimmune disorder patients after string-matching AD terms to concepts (Table S7) and checking for billing concept occurrence in each patient record in the UCSF and Stanford systems. We only included AD billing concepts related to late-onset or sporadic AD in our search, as early-onset AD is thought to have distinct etiology and stronger genetic components.^51^ However, some individuals with early onset (<65 years) likely did make it into our study groups as a result of certain non-specific AD billing codes, so we ran a sensitivity analysis removing individuals <65 years old (see results section and sensitivity analyses section in STAR Methods for more details). Additional demographic data from the UCSF and Stanford EHR systems was collected on patients including date of birth, date of death (if available), self-reported race, self-reported ethnicity, and sex at birth. We also computed healthcare utilization statistics on patients, including number of doctor’s visits, total number of unique diagnoses, and first and last medical visit date.

We identified healthy control individuals without autoimmune disorders to compare to the autoimmune patient groups from the UCSF and Stanford databases. To do this, we searched for patients *without* any of the 878 final autoimmune disorder billing concepts present in their records, and we additionally removed individuals from the healthy control group who had concepts that were similar to any of the 878 final autoimmune concepts. For example, an individual who had the billing concept “family history of Celiac disease” in their EHR but did not have a more specific Celiac disease billing concept identifying a personal diagnosis of Celiac disease would have been excluded. Similarly, several billing concepts that were too general to pertain specifically to an autoimmune disorder (e.g., kidney disease) but that represented a serious condition were excluded from the healthy controls. For our cohort study design, we matched the healthy controls to autoimmune disorder patients based on criteria further described in the Study Group Matching and Quality Control section of the Methods.

To identify a population of non-AD healthy controls to compare to our AD patient group, we searched for patients *without* any AD billing concepts in their records and matched them with AD cases based on demographic factors. To clarify some terminology, the control individuals that were matched to autoimmune disorder cases will be referred to as the “non-autoimmune controls” going forward, whereas the control individuals that were matched to AD cases will be referred to as the “non-AD controls”. These are two separate groups of controls, but they may have overlap in individuals as someone without both an autoimmune disorder and AD might be in both control groups. Additionally, someone in one of the disease groups may be in a control group for the other disease. For example, an AD patient might show up in the non-autoimmune control group, since the requirement to be in that group is not having an autoimmune disorder.

#### Sex as a biological variable

Sex-at-birth is reported in both the UCSF and Stanford OMOP EHR systems. We refer to it as sex for brevity throughout the manuscript. We accounted for sex as a biological variable by performing all analyses in female- and male-specific subsets of our original study groups. This allowed us to test if any risk associations differed by sex, and if autoimmunity subtypes or individual disorders were driving AD risk associations in a particular sex. We filtered out individuals with an unknown sex from our UCSF study groups. Due to differences in encoding sex within the UCSF and Stanford EHR systems, there were a small number of individuals (0.02% of the total) with an unknown sex that were included in the Stanford cohort dataset. These individuals were not included in any sex-specific analyses.

### Method details

#### Data cleaning and quality control

Our data cleaning pipeline involved several steps. First, we performed quality control to remove any individuals with missing demographic information in the self-reported race, self-reported ethnicity, sex, and birth year categories from consideration for our disease and healthy control groups in the UCSF dataset. Due to differences in encoding some of this demographic information between UCSF and Stanford EHR systems, a small number of individuals with unknown demographic values were included in the Stanford dataset (see Sex as a Biological Variable), but each individual with an “unknown” demographic field was similarly matched with another Stanford individual with an “unknown” value, removing any issues comparing people without matching information. In the UCSF study groups, we also required individuals to have a valid reported age at death, as this allowed us to compare individuals by total lifespan. We restricted individuals to be between 30 and 90 years of age at their death in these groups. In the Stanford study groups, there was a substantial amount of censored death information such that including lifespan information in matching would have extremely limited our study group sizes, so we did not enforce this constraint. We verified that leaving out lifespan as a matching variable in the Stanford groups did not significantly alter overall risk signals, so we felt confident leaving it out of our validation study group criteria (See sensitivity analyses support risk associations between autoimmune disorders and AD in results). It is likely that, because of censored data being included in the Stanford datasets, the increased risk associations we saw in our analyses would be even stronger (see discussion). In addition to quality control on demographics, we also removed individuals who had zero hospital visits or whose first and last visit dates were the same.

We next determined: 1) who in the autoimmune disorder patient groups and corresponding non-autoimmune control groups had an AD diagnosis, and 2) who in the AD patient groups and corresponding non-AD control groups had an autoimmune disorder diagnosis. Within our autoimmune/AD disease groups and respective healthy control groups, different individuals were demarcated with their assigned “person ID” following OMOP conventions. We then determined which person IDs of one group overlapped with the person IDs of another group. For example, to discover which autoimmune patients and non-autoimmune controls had an AD diagnosis, we determined which of the person IDs of our AD patient group overlapped with the person IDs of the autoimmune and non-autoimmune groups. In a similar manner, we determined which AD patients and which non-AD controls had an autoimmune diagnosis by overlapping the person IDs of our autoimmune patient group with the AD and non-AD person IDs.

We also identified the relative dates of AD and autoimmunity diagnoses for patients. To focus on the effect of autoimmunity on AD, we did not consider individuals who had an AD diagnosis prior to their autoimmune disorder diagnosis. Specifically in the UCSF dataset, we computed several metrics for each individual to aid the matching of autoimmune or AD patients to their respective controls when performing different sensitivity analyses. These metrics included each individual’s age when they died, the total length of the UCSF EHR record (last visit date - first visit date), and the frequency of doctor’s visits per year.

To understand which autoimmune disorder subtypes might be driving AD risk, we grouped the 26 autoimmune disorders of interest into 8 distinct subtype categories (Table S1 and Figure 3A) and assigned patients into categories based on which autoimmune disorder(s) they had. If a patient had multiple autoimmune disorders (UCSF *n* = 2,378 patients, Stanford *n* = 37,332 patients) across different subtype categories, they were counted once within each subtype risk association analysis, and therefore could be represented in more than one analysis.

#### Study group matching and quality control

To mitigate selection bias and ensure robustness of results, we examined the risk of receiving an AD diagnosis following an autoimmune disorder diagnosis using two study designs in each of our EHR datasets: a retrospective case-control study with AD patients and non-AD matched controls, and a retrospective cohort study with autoimmune disorder patients and non-autoimmune matched controls. We performed 1:1 matching of patients to controls for each study group using propensity scoring on each individual’s birth year, sex, self-reported race, and self-reported ethnicity (Figures S6, S7, S8 and S11–S13). We also matched on lifespan in the UCSF study groups (See Data Cleaning and Figures S6 and S7). We enforced exact matches between patients and controls in the categories of sex, self-reported race, and self-reported ethnicity. In our main analysis and throughout follow-up sensitivity analyses, we ensured high-quality matching by verifying that the average absolute standardized mean error between each matched pair was less than 0.1, which is considered a good match throughout the literature.^52^ Matching statistics for the main risk analysis are reported in Figures S6, S7, S8, and S11–S13. We conducted final quality control in both study groups of our main and sensitivity analyses by removing any matched pairs of individuals where a control individual was diagnosed with AD prior to the matched disease case being diagnosed with an autoimmune disorder, to eliminate some aspects of reverse causality. After matching and cleaning, we were left with four “study groups”: a case-control and cohort study group from UCSF, and a case-control and cohort study group from Stanford. Study group characteristics are listed in Table 1.

#### Chart review

Given the misclassification bias that can be present in EHRs, we performed a chart review on 50 randomly selected individuals from the UCSF study groups to determine whether these people were truly AD patients or non-AD controls. We extracted the individuals’ notes from the UCSF EHR database and worked with collaborating clinicians to determine whether the patient outcomes observed from the EHR billing codes aligned with what was in the unstructured clinical notes. We used three different categories to indicate our confidence that each note represented a true AD diagnosis: the individual’s clinical notes indicate a clear AD diagnosis, the individual’s clinical notes clearly indicate lack of an AD diagnosis, and the individual’s notes indicate a dementia diagnosis but are unclear as to whether the dementia subtype is AD. We first report classification statistics in the main text on those patients (*N* = 40) that we were able to definitively classify with respect to AD. Under these conditions, PPV was 0.82, negative predictive value (NPV) was 1, sensitivity was 1, and specificity was 0.94. Half (5/10) of the inconclusive dementia individuals were classified as having AD based on billing codes and half were not. Many forms of dementia exist other than AD, and it is likely that these individuals truly contain a mix of AD and non-AD dementias. If we treat all these individuals as not having AD or as having AD, the PPV ranges between 0.6 and 0.9, the NPV between 0.85 and 1.0, and sensitivity between 0.74 and 1.0. Further chart review statistics are in Table S10.

#### Risk analysis

To enable comparison across the different study designs, we computed odds ratios to quantify the risk of being diagnosed with AD in autoimmune disorder patients compared to non-autoimmune controls. Across each of the study groups, we computed odds ratios at three levels: 1) across all autoimmune disorders combined, 2) across autoimmunity subtypes, and 3) across individual autoimmune disorders. At each of these three levels, we repeated the analysis in a sex-stratified manner to explore if risk was sex-specific. All odds ratios were computed using Fisher’s exact test on contingency tables of AD/autoimmunity patients.

#### Prevalence analysis

We calculated the prevalence of AD in patients with autoimmune disorders compared to matched controls in a sex-stratified manner in the cohort study groups. To ensure that a difference in prevalence was not due to underlying demographic differences between female and male individuals, we took the cohort study groups across UCSF and Stanford and matched the smaller sample size of male individuals to female individuals based on birth year, self-reported race, and self-reported ethnicity, while matching additionally on lifespan in the UCSF study groups. Again, we used exact matching on self-reported race and self-reported ethnicity while using propensity score matching on the remaining variables. We then calculated the percentage of people with AD in each stratification: female individuals with autoimmune disorders, male individuals with autoimmune disorders, female non-autoimmune control individuals, and male non-autoimmune control individuals. To obtain a 95% confidence interval for each prevalence statistic, we bootstrapped the data 1,000 times. We again used a Fisher’s exact test to compute significance of prevalence differences among stratifications.

#### Sensitivity analyses

We report on several sensitivity analyses that we conducted to show the robustness of our results to many EHR confounders and epidemiological confounders. Matching statistics for many of the covariates that we added to our matching pipeline to account for confounding are shown in Figures S9 and S10, and matching among demographic variables remained high-quality (SMD<0.1) throughout each sensitivity analysis, as in the main risk analysis. Specifically for the comorbidity sensitivity analysis, we used the Charlson-Deyo Comorbidity Index (CDCI)^22^^,^^23^ to identify chronic conditions that could potentially influence the risk associations we saw between autoimmunity and AD. This index includes 17 categories of comorbidities, two of which are dementias and rheumatic diseases. As these were our two categories of interest for the study, we did not include these in our comorbidity analysis, leaving us with 15 classes of comorbidities to investigate. The CDCI is based on ICD-10 codes, and we mapped these to standardized OMOP codes in the UCSF or Stanford OMOP databases to identify which individuals had which comorbidities. The ICD-10 codes we used and their corresponding mapped standardized codes are listed in Table S9. We matched individuals on the presence or absence of each comorbidity category at UCSF and Stanford, with an additional analysis where individuals were matched on the total number of comorbidity categories each individual had present in their record at UCSF. Results of these sensitivity analyses are listed in Table S4.

#### Longitudinal AD onset analysis

To understand the effect of autoimmunity on the risk of AD diagnosis over time, we conducted a longitudinal age of onset analysis. For this, we constructed new longitudinal study groups from the UCSF and Stanford EHR systems, starting by selecting individuals with both an autoimmune disorder and AD from our cohort study groups. We matched the autoimmunity patients with AD to individuals without autoimmune conditions who had AD from the background UCSF and Stanford EHR databases. The same variables used to match individuals for the main odds ratio analysis (birth year, age at death when available, sex, race, and ethnicity) were also used to match the time-to-event study groups here. These time-to-event cohorts also passed our data quality control pipeline described previously. The age of AD onset for each individual in each longitudinal study group was then calculated by taking the difference in time between a person’s birth year and the first appearance of an AD billing concept in the person’s medical record. We used a Mann-Whitney U test and Cox proportional hazard modeling to compare age distributions and the rate of AD diagnosis over time.

### Quantification and statistical analysis

Matching to construct study groups was performed using the matchit function of the MatchIt package (version 4.5.3). The nearest neighbor method was used, and exact matching on self-reported race, self-reported ethnicity, and sex at birth was used as a default unless otherwise stated. All Fisher’s exact tests to compute risk and prevalence statistics were conducted using the oddsratio.fisher function of the epitools package (version 0.5–10.1). We also used the tableone package (version 0.13.2) to generate demographic tables. Finally, Mann-Whitney U tests and Cox proportional hazard modeling was performed using the stats (version 4.1.3) and survival/survminer (version 3.5–5/0.4.9) packages, respectively. We conducted all computational steps using R (version 4.1.3), and plots were created with the ggplot2 package (v3.4.2).

Statistical details of analyses, such as sample sizes and *p* values, can be found in figures and the main text, as well as in Table S8 containing all data points represented in plots. To account for multiple hypothesis testing, we used Bonferroni corrections for each odds ratio analysis. For the disease subtype risk analysis, we corrected *p*-values within each overall or sex-stratified group (e.g., a correction factor of 8 for the 8 disease subtype comparisons). For the specific disorder risk analysis, we performed a within-disorder-subtype *p*-value correction to evaluate which specific conditions within an autoimmune disorder subtype group had significant subtype effects. This meant the correction factor for a particular disorder comparison was determined by the number of conditions within that particular disorder’s subtype category (e.g., *p*-values for each autoimmune disorder in the endocrine subgroup were corrected by a factor of 4, due to the endocrine subgroup being comprised of 4 diseases).

## Supplemental information

Document S1. Figures S1–S13

Table S1. Excel file containing autoimmune disorder prevalence statistics in each study group, related to STAR Methods and Figure 3

Table S2. Excel file containing risk ratio statistics for odds ratio comparisons, related to STAR Methods

Table S3. Excel file containing statistical power calculations for each analysis, related to Figures 2–4 and Table S4

Table S4. Excel file containing risk analysis results from several sensitivity analysis conditions, related to STAR Methods and Figures 2 and 3

Table S5. Excel file containing string autoimmune disease terms used to identify the billing concept codes in Table S6, related to Figure 1

Table S6. Excel file containing medical billing concept codes used to identify individuals with autoimmune disorders, related to Figure 1

Table S7. Excel file containing medical billing concept codes used to identify individuals with AD, related to Figure 1

Table S8. Excel file containing main figure data points, related to Figures 2 and 3

Table S9. Excel file containing medical billing concept codes used to identify comorbidities of interest, related to STAR Methods

Table S10. Excel file containing chart review statistics, related to STAR Methods

Document S2. Article plus supplemental information
