# Supplementary material for: Exposure to autoimmune disorders is associated with increased Alzheimer’s disease risk in a multi-site electronic health record analysis
Source: Cell Rep Med. 2025 Feb 24;6(3):101980. doi: 10.1016/j.xcrm.2025.101980 (PMC11970322; doi:10.1016/j.xcrm.2025.101980)
Supplement: Document S1. Figures S1–S13 [file mmc1.pdf]

Cell Reports Medicine, Volume *6*

Supplemental information

Exposure to autoimmune disorders is associated with increased Alzheimer's disease risk in a multi-site electronic health record analysis

Grace D. Ramey, Alice Tang, Thanaphong Phongpreecha, Monica M. Yang, Sarah R. Woldemariam, Tomiko T. Oskotsky, Thomas J. Montine, Isabel Allen, Zachary A. Miller, Nima Aghaeepour, John A. Capra, and Marina Sirota

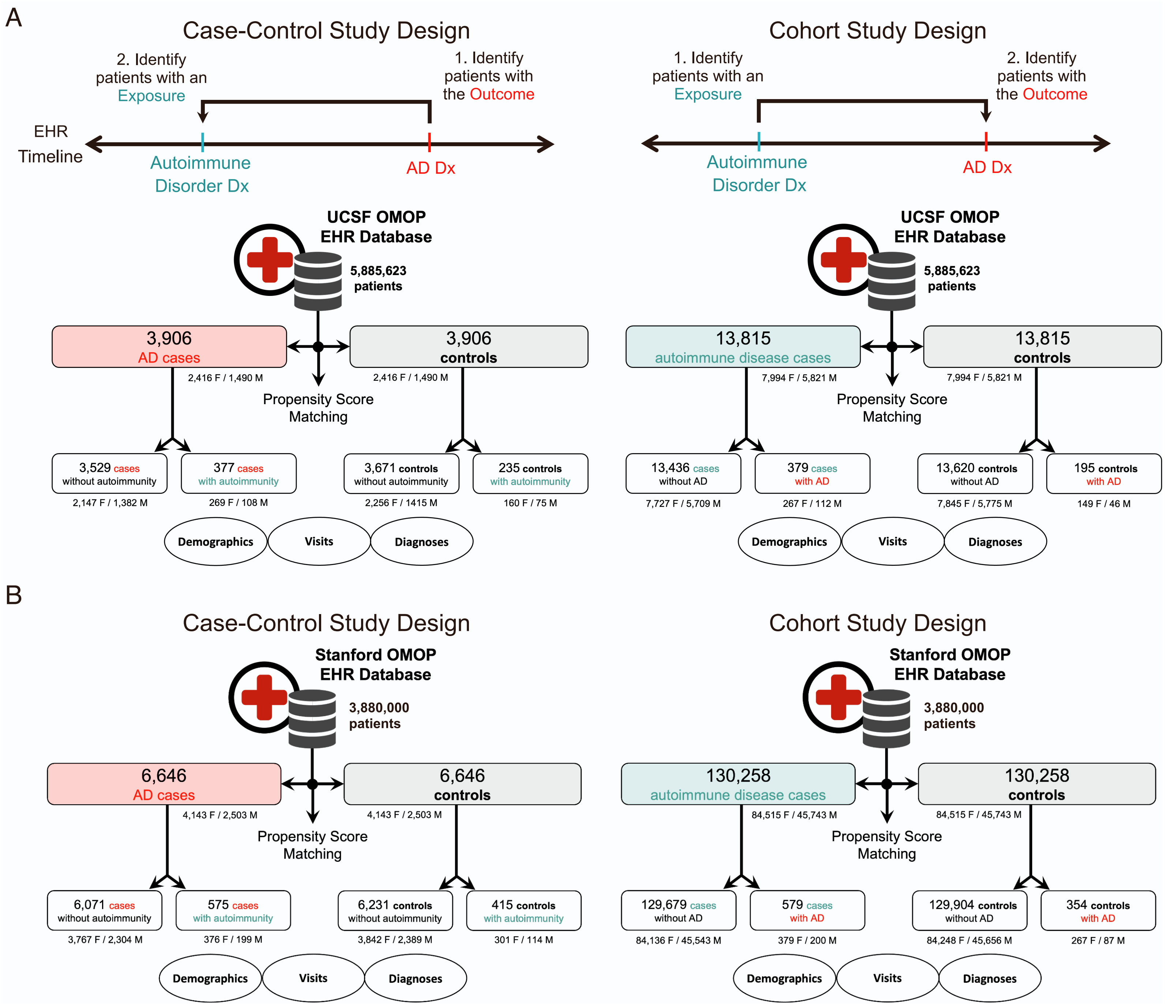

Figure S1: Full breakdown of study groups in UCSF and Stanford data sets. Related to Figure 1. Numbers of patients with autoimmune disorders and/or AD in the case-control (left) and cohort (right) study groups of either the UCSF (A) or Stanford (B) data sets. Also pictured are the breakdowns of each category by sex. Dx = Diagnosis, M = Male, F = Female.

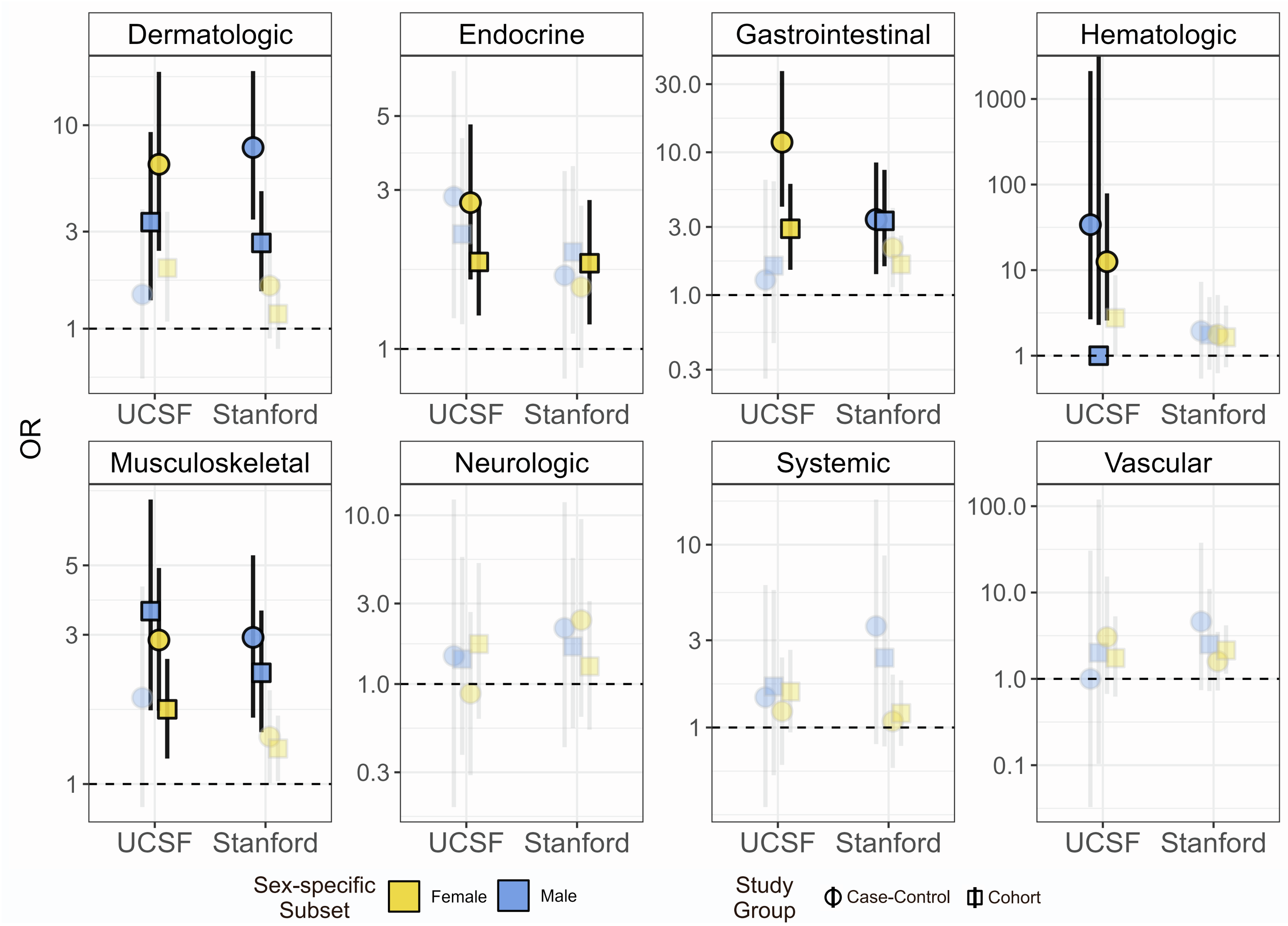

Figure S2: Autoimmune disorder subtypes exhibit varying sex-specific AD risk effects. Related to Figure 3. AD odds ratios for each of the eight autoimmune disorder subtypes across female- and male-specific subsets of each EHR system and study group. Significant odds ratios are bold/outlined in black while insignificant odds ratios are faded/more transparent. Many subtypes associated with increased AD risk predominantly in one sex more than another (e.g., dermatologic disorders in males and endocrine disorders in females), whereas other subtypes were less clear in their sex-specific effects (e.g., gastrointestinal and musculoskeletal conditions), which varied between discovery and validation data sets.

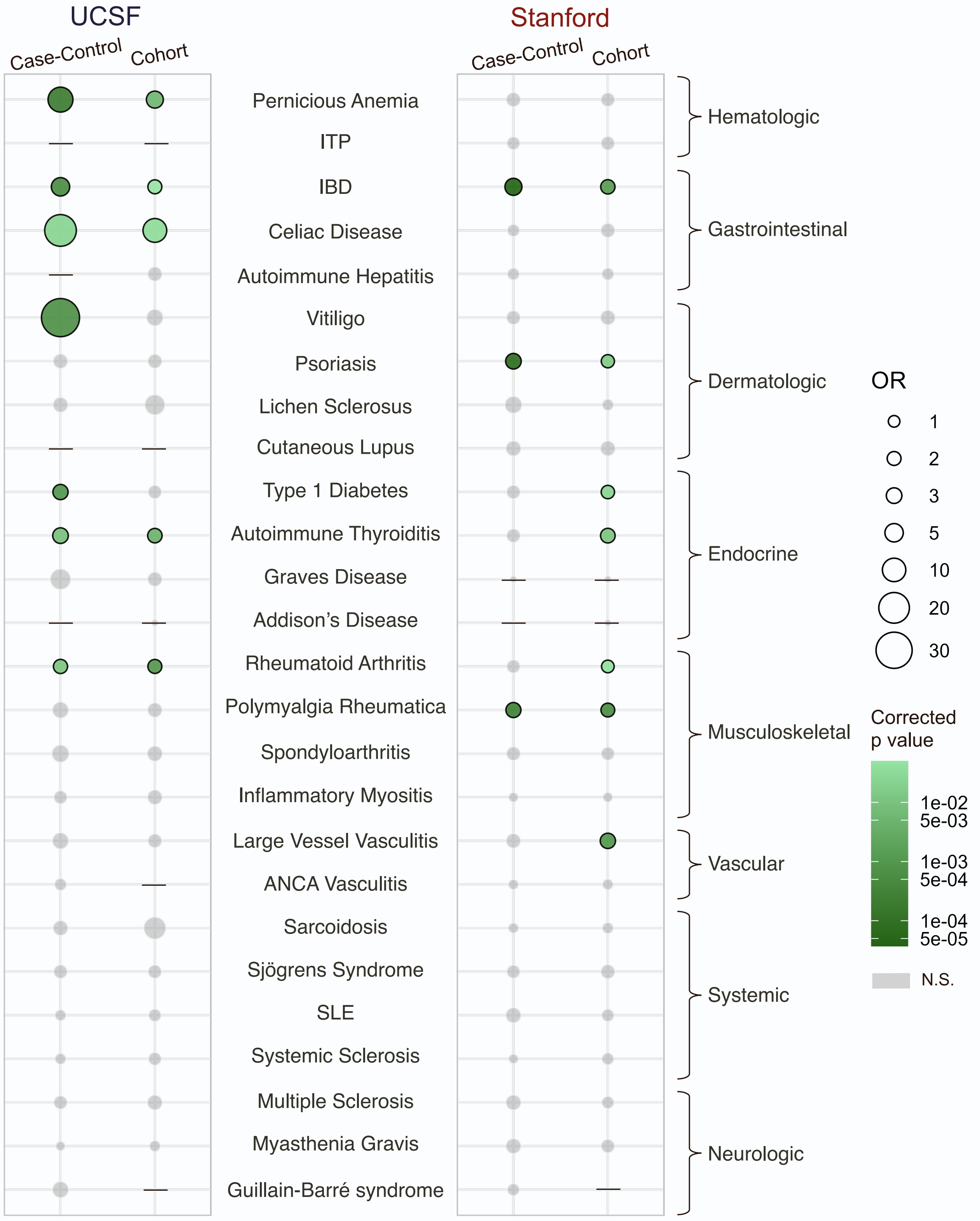

Figure S3: Specific autoimmune disorders are associated with increased AD risk. Related to Figure 3. AD odds ratios for each of the 26 specific autoimmune disorders we analyzed. Many were significant across several study designs and data sets, including IBD, type 1 diabetes, autoimmune thyroiditis, and rheumatoid arthritis. For several of the more rare autoimmune disorders, we were underpowered to detect risk associations, and those with sample sizes that were too small for calculations (e.g., zero AD cases) or resulted in uninterpretable estimations (e.g., infinite odds ratios or confidence interval estimations) are denoted with black horizontal line segments. These diseases are nonetheless interesting and warrant more research. ITP = Idiopathic Thrombocytopenic Purpura, IBD = Inflammatory Bowel Disease, SLE = Systemic Lupus Erythematosus, N.S. = Not Significant.

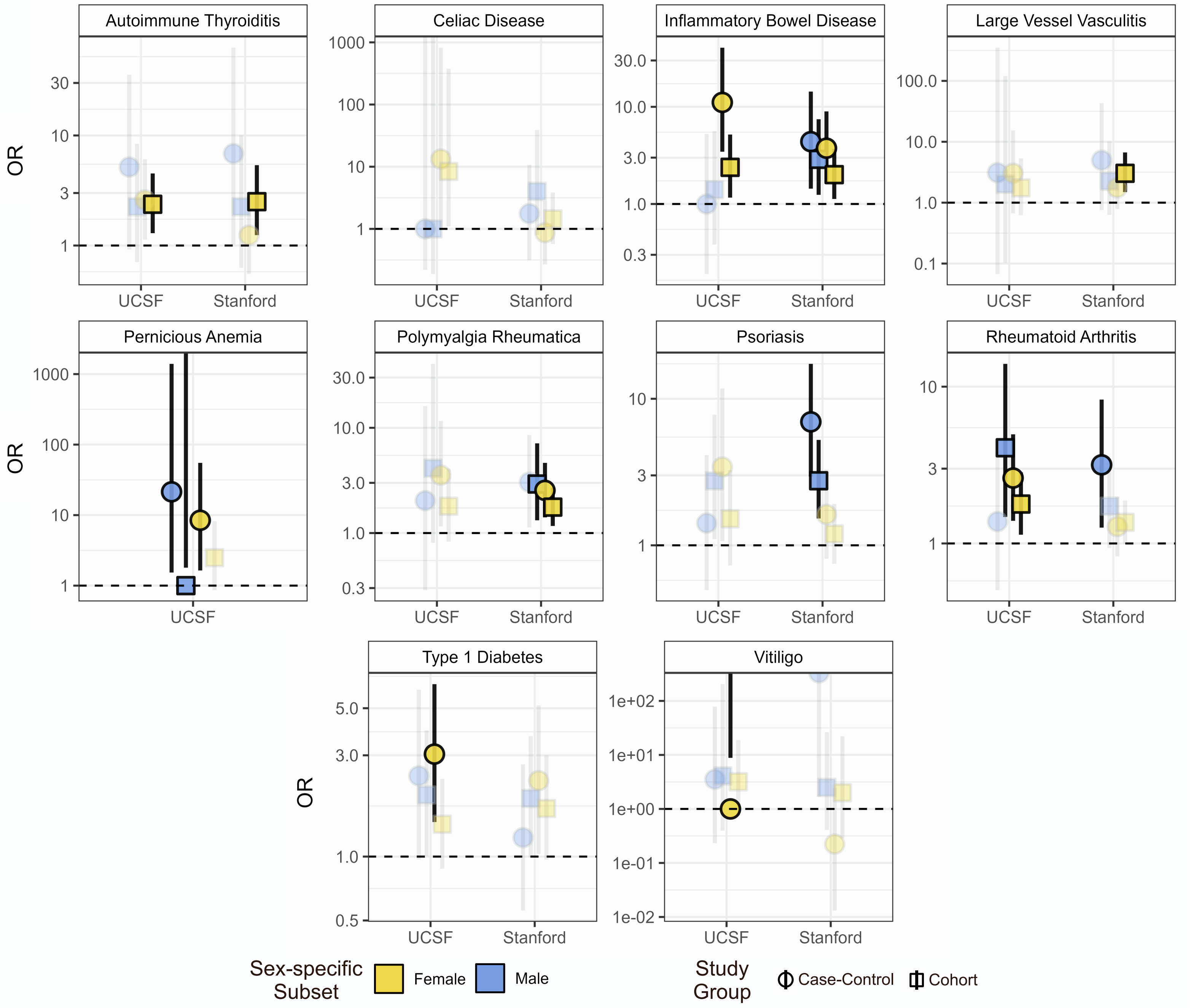

Figure S4: Increased AD risk from specific autoimmune disorders is potentially sex-specific. Related to Figure 3. Sex-specific AD odds ratios for each of the specific autoimmune disorders that were significant across multiple data sets in the overall analysis (Fig 3B). Significant odds ratios are outlined in black while insignificant associations are faded/more transparent. Autoimmune thyroiditis was associated with increased risk primarily in women, potentially driving the female-specific AD risk association for endocrine diseases (Fig S2). Given that we were underpowered to detect several of these associations after stratifying, more research into the sex-specific effects of particular autoimmune disorders is warranted, especially given the sex disparities that remain despite increased risk across both sexes overall. Odds ratios pictured that fall outside of their respective confidence intervals were biologically uninterpretable due to infinitely high confidence intervals and/or odds ratios.

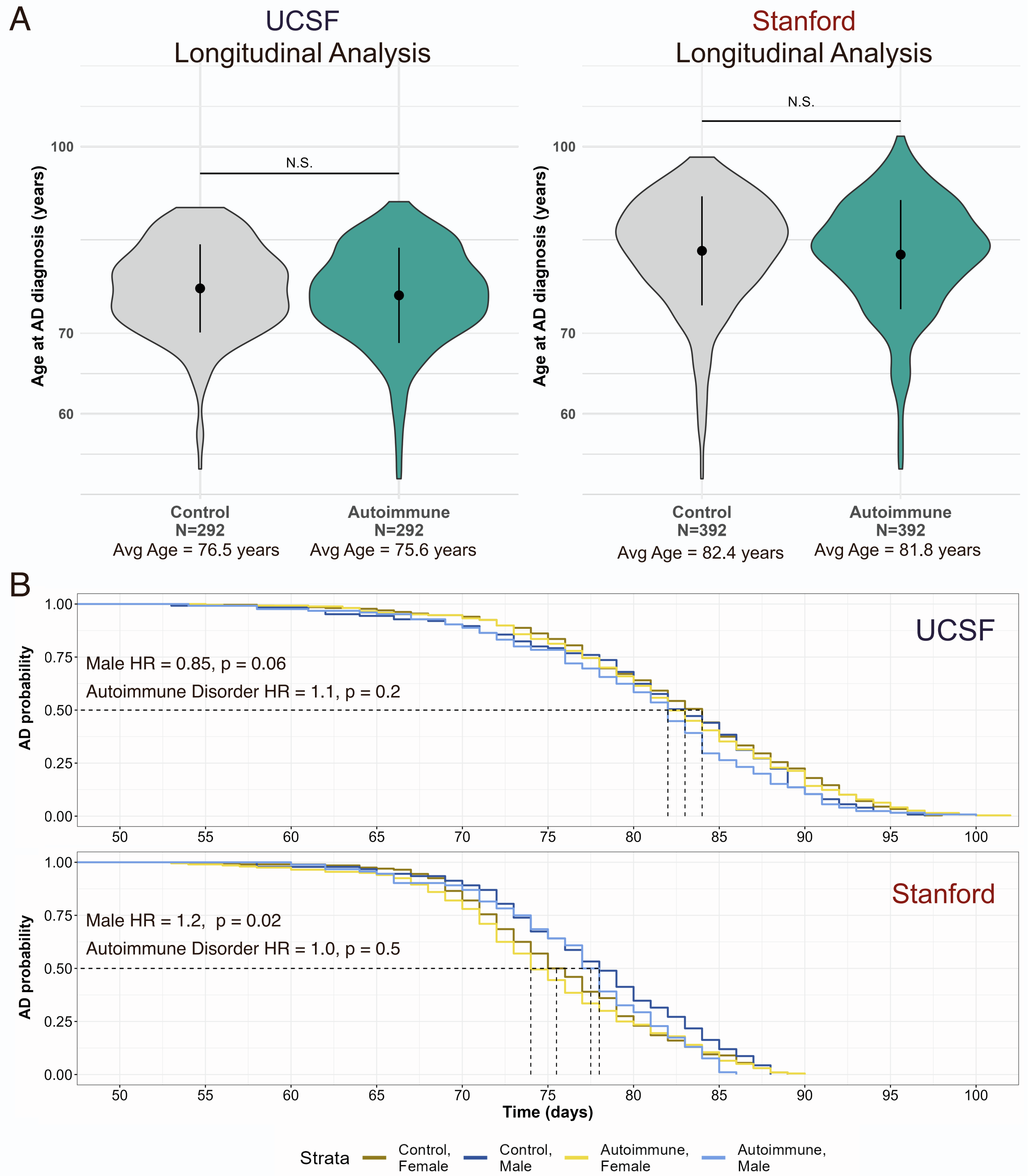

Figure S5: Autoimmune disorders trend toward accelerating AD onset. Related to Figure 4. (A) Distributions of AD diagnosis age among individuals with autoimmune disorders and non-autoimmune controls in UCSF (left) and Stanford (right) longitudinal cohorts. While younger age of onset in autoimmune patients was insignificant compared to controls, likely due to power, there was about a one-year difference between autoimmune patients and non-autoimmune controls. (B) Survival curves for individuals in the longitudinal study groups at UCSF (top) and Stanford (bottom). Like in the distributional analysis (Figure 4B), sex tended to separate individuals into groups of relatively earlier or later AD onset.

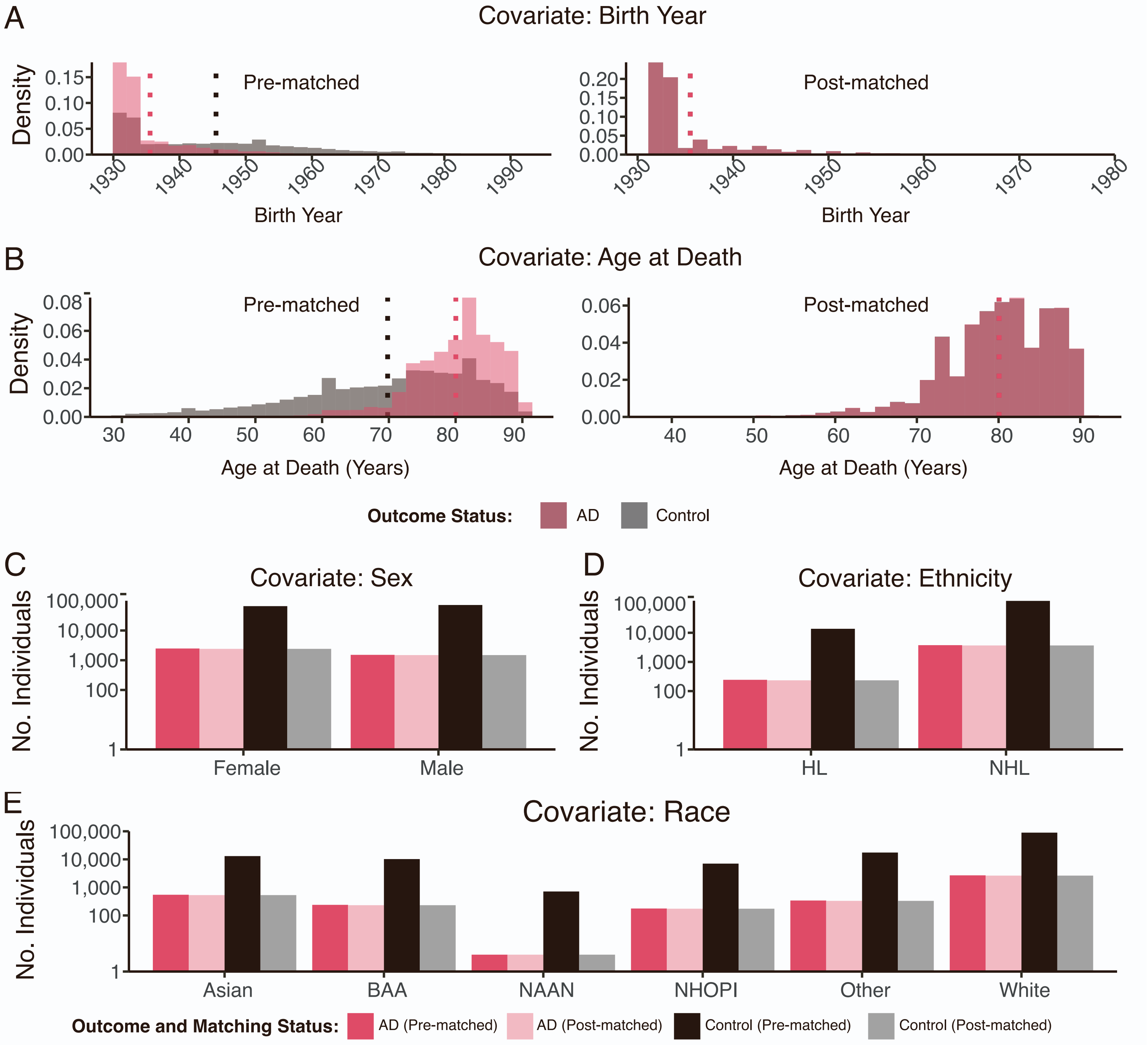

Figure S6: UCSF Case-Control Matching Statistics. Related to Figure 1. Matching statistics for the main analysis in the UCSF case-control study group (further matching statistics for each sensitivity analysis are reported in later supplementary figures and in the supplementary tables). (A) Distribution of birth years among AD patients and non-AD controls prior to (left) and after (right) matching. Dotted lines represent the mean of each outcome subgroup. (B) Same as A but for age at death among individuals. (C) Number of female and male individuals in pre- and post-matched groups of AD and non-AD individuals. (D), (E) Same as C for self-reported ethnicity and race categories, respectively. HL = Hispanic/Latino, NHL = Not Hispanic/Latino, BAA = Black or African American, NAAN = Native American or Alaska Native, NHOPI = Native Hawaiian or Other Pacific Islander.

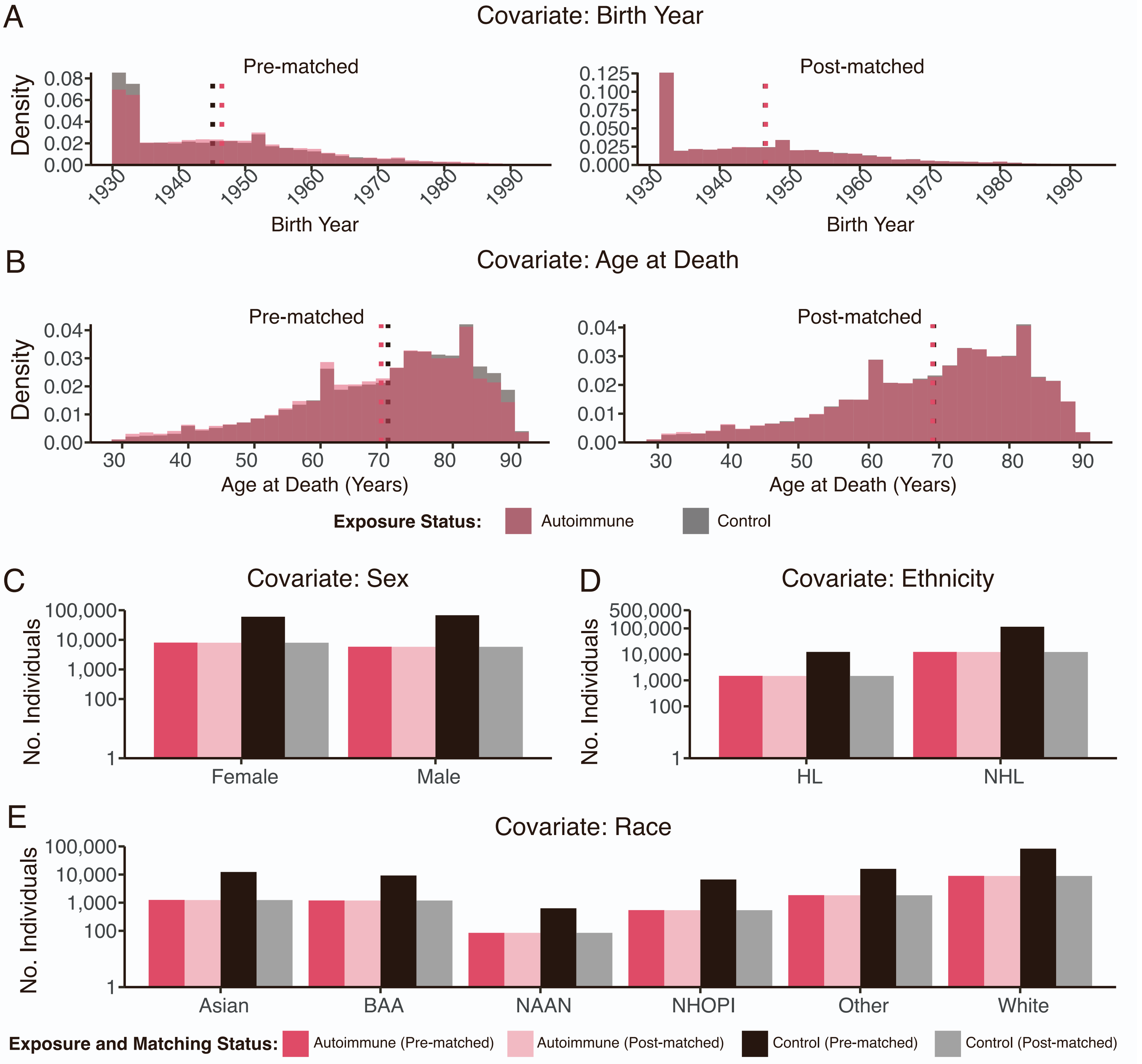

Figure S7: UCSF Cohort Matching Statistics. Related to Figure 1. Matching statistics for the main analysis in the UCSF cohort study group (further matching statistics for each sensitivity analysis are reported in the supplementary tables. (A) Distribution of birth years among autoimmune patients and non-autoimmune controls prior to (left) and after (right) matching. Dotted lines represent the mean of each exposure subgroup. (B) Same as A but for age at death among individuals. (C) Number of female and male individuals in pre- and post-matched groups of exposed and unexposed individuals. (D), (E) Same as C for self-reported ethnicity and race categories, respectively. HL = Hispanic/Latino, BAA = Black or African American, NAAN = Native American or Alaska Native, NHOPI = Native Hawaiian or Other Pacific Islander.

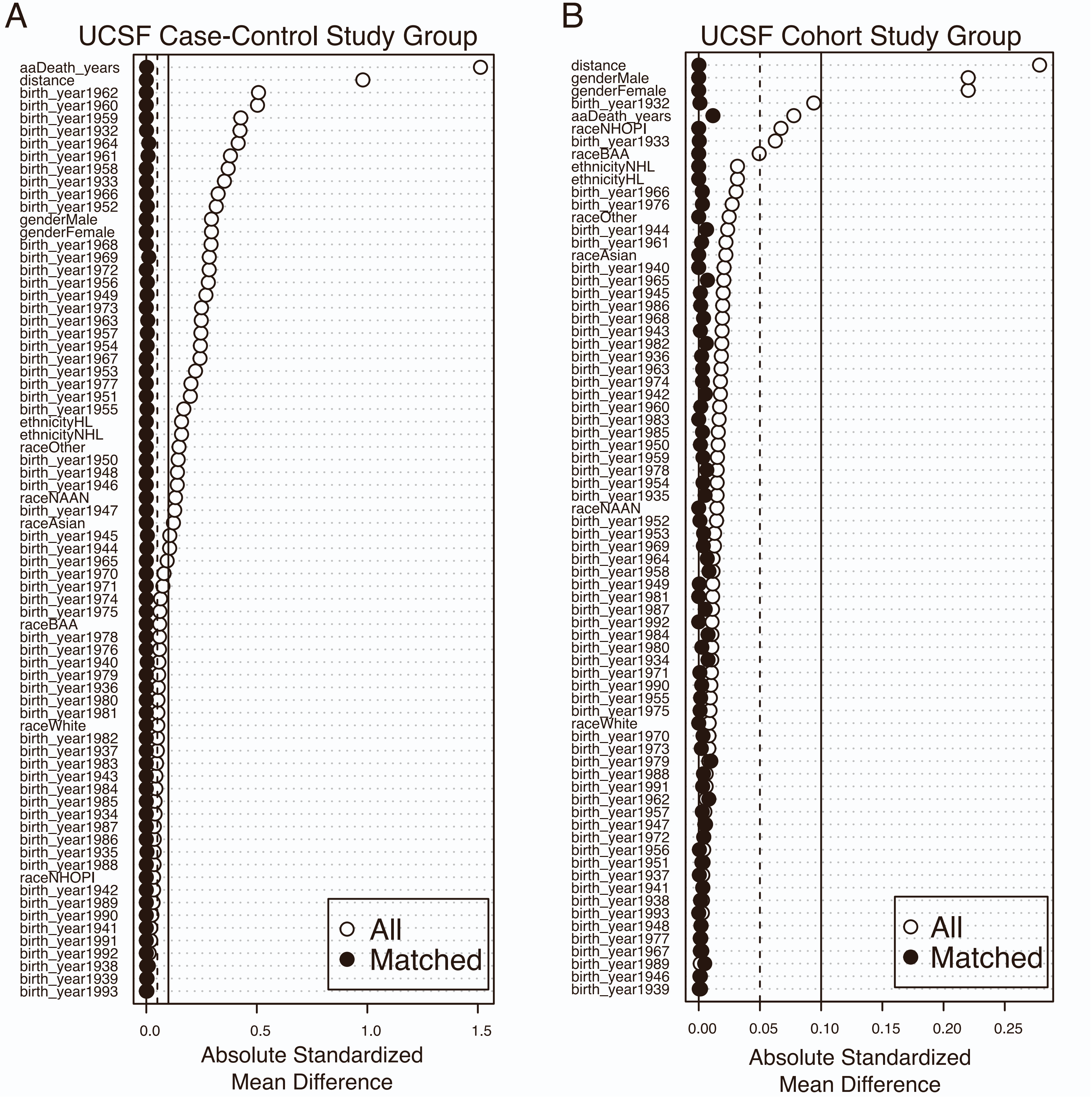

Figure S8: UCSF Matching Distances. Related to Figure 1. Absolute standardized mean differences for covariates that were used for matching in the main UCSF risk analysis, for either the case-control (A) or cohort (B) study group. Covariate distances for other sensitivity analyses are reported in other supplementary figures and tables. HL = Hispanic/Latino, NHL = Not Hispanic/Latino, BAA = Black or African American, NAAN = Native American or Alaska Native, NHOPI = Native Hawaiian or Other Pacific Islander.

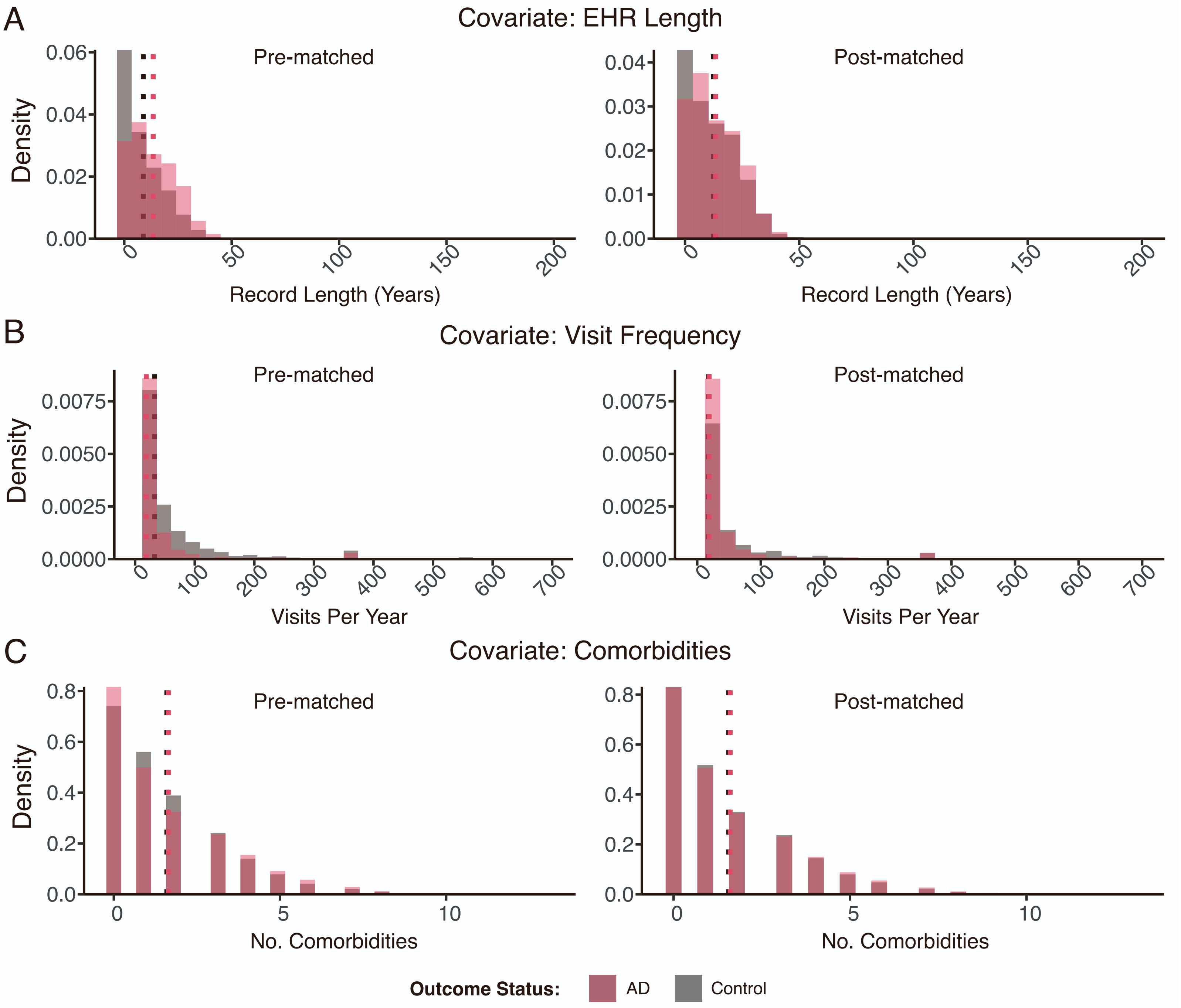

Figure S9: Sensitivity Analysis Covariate Distributions in the UCSF Case-Control Study Group. Related to Figure 1. (A) Length of each individual's EHR record at UCSF prior to (left) and after (right) matching in the AD and non-AD groups. Record length was truncated at 200 years. Longer times could potentially represent inaccuracies in the EHR record. (B) Same as A but for visit frequency for each individual before and after matching. Visits per year were truncated at 700 along the x axis for ease of viewing. (C) Same as A but for number of comorbidity categories present in each individual. An individual could have a maximum of 15 comorbidities given the 15 comorbidity categories we used in our sensitivity analysis.

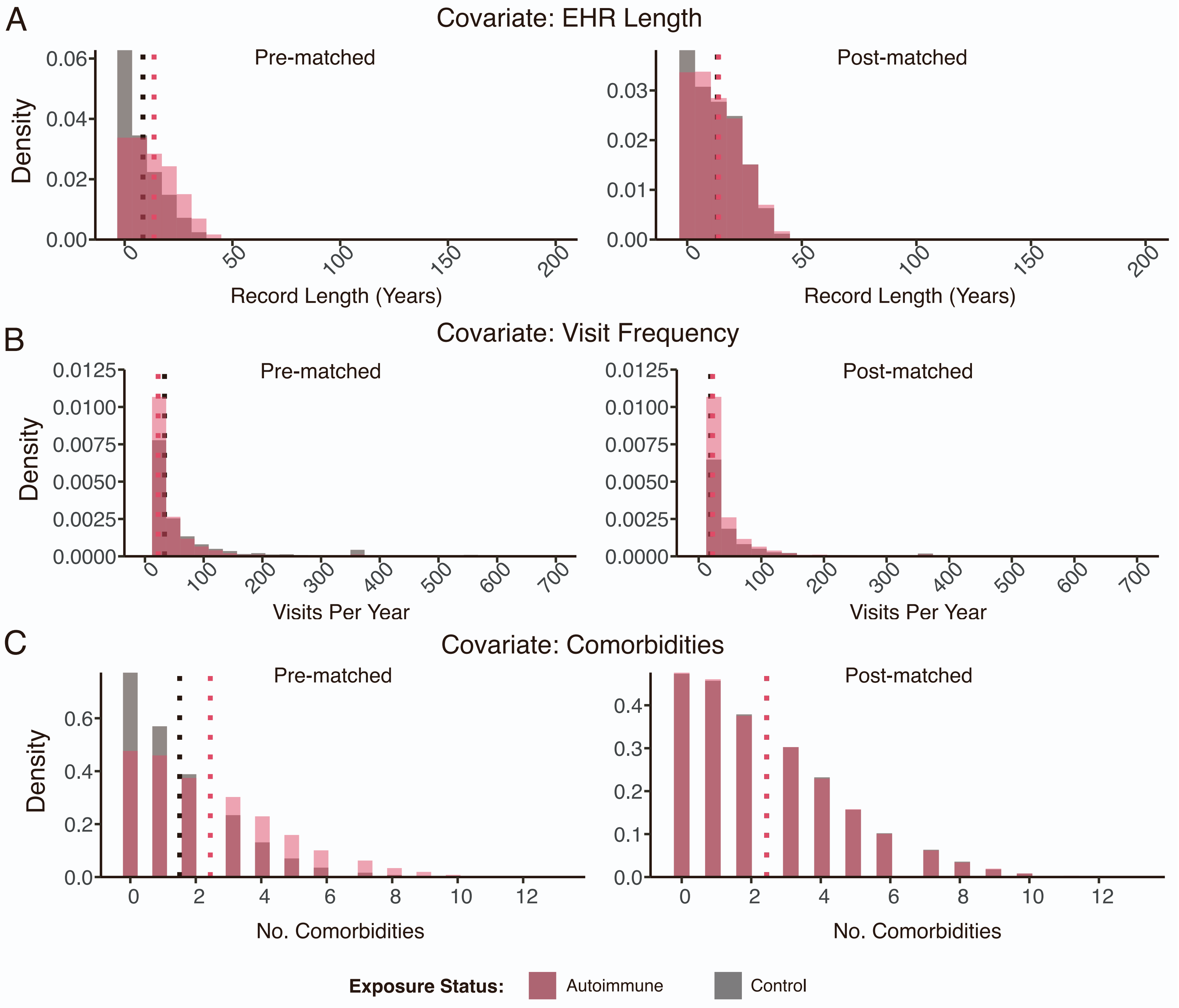

Figure S10: Sensitivity Analysis Covariate Distributions in the UCSF Cohort Study Group. Related to Figure 1. (A) Length of each individual's EHR record at UCSF prior to (left) and after (right) matching in the exposed and unexposed groups. Record length was truncated at 200 years. Longer times could potentially represent inaccuracies in the EHR record. (B) Same as A but for visit frequency for each individual before and after matching. Visits per year were truncated at 700 along the x axis for ease of viewing. (C) Same as A but for number of comorbidity categories present in each individual. An individual could have a maximum of 15 comorbidities given the 15 comorbidity categories we used in our sensitivity analysis.

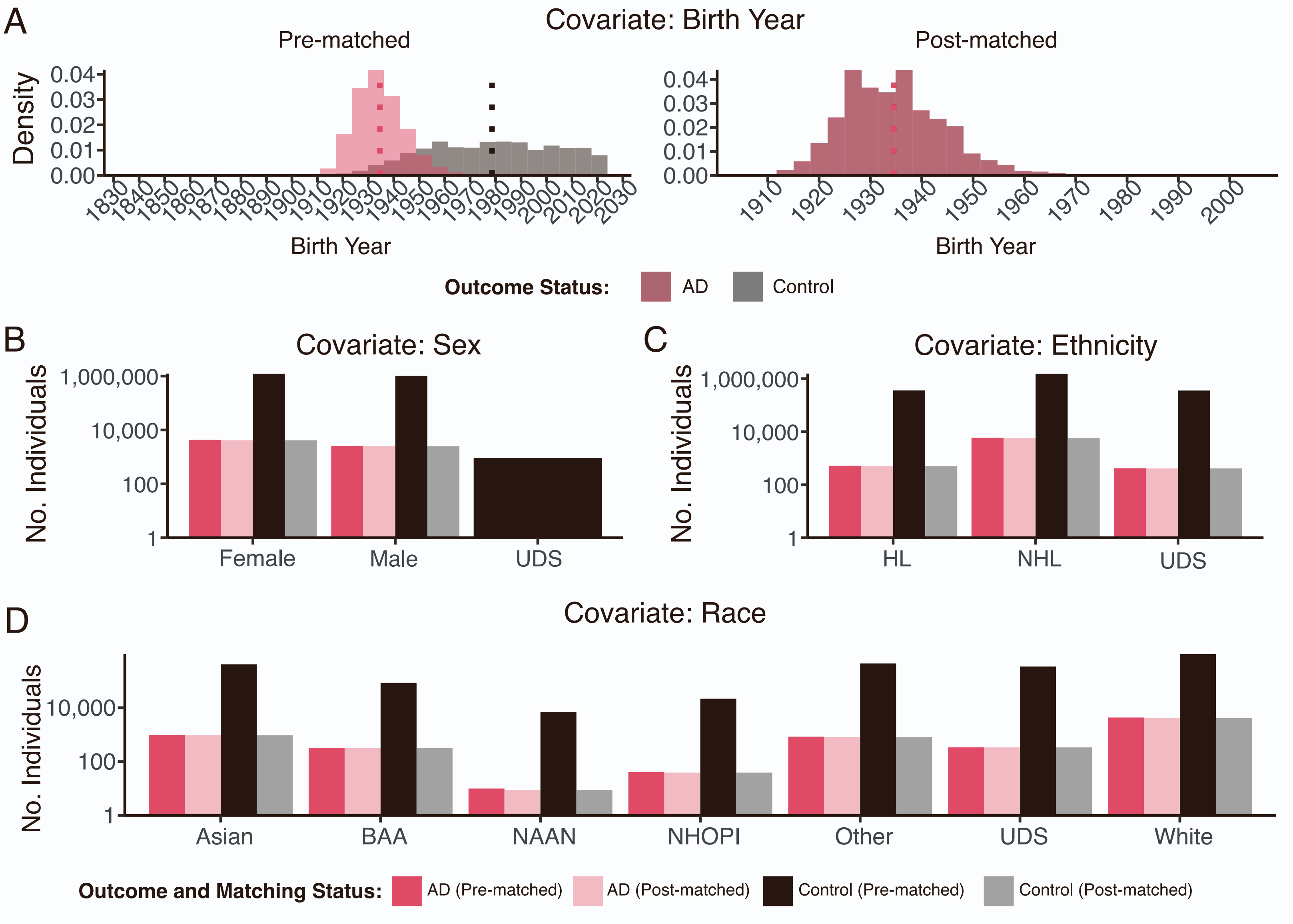

Figure S11: Stanford Case-Control Matching Statistics. Related to Figure 1. Matching statistics for the main analysis in the Stanford case-control study group. (A) Distribution of birth years among AD patients and non-AD controls prior to (left) and after (right) matching. Dotted lines represent the mean of each outcome subgroup. (B) Number of female and male individuals in pre- and post-matched groups of AD and non-AD individuals. Note that the UDS category is a uniform color because no individuals from this category made it into the final matched study group. (C) Same as B for self-reported ethnicity categories. (D) Same as B for self-reported race categories. HL = Hispanic/Latino, NHL = Not Hispanic/Latino, BAA Black or African American, NAAN = Native American or Alaska Native, NHOPI = Native Hawaiian or Other Pacific Islander, UDS = Unknown/Decline to State.

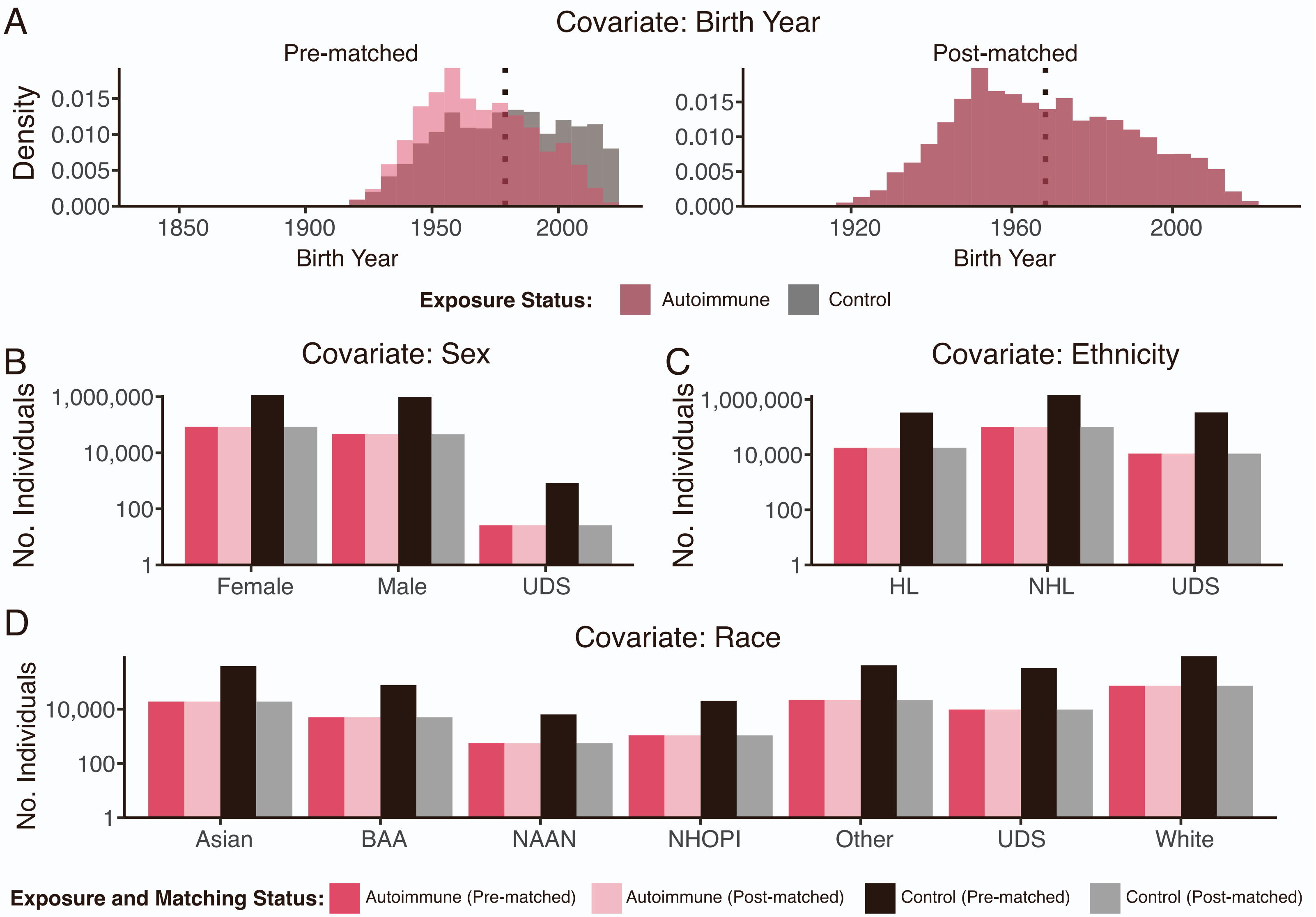

Figure S12: Stanford Cohort Matching Statistics. Related to Figure 1. Matching statistics for the main analysis in the Stanford cohort study group. (A) Distribution of birth years among autoimmune patients and non-autoimmune controls prior to (left) and after (right) matching. Dotted lines represent the mean of each exposure subgroup. (B) Number of female and male individuals in pre- and post-matched groups of exposed and unexposed individuals. (C) Same as B for self-reported ethnicity categories. (D) Same as for B for self-reported race categories. HL = Hispanic/Latino, NHL = Not Hispanic/Latino, BAA = Black or African American, NAAN = Native American or Alaska Native, NHOPI = Native Hawaiian or Other Pacific Islander, UDS = Unknown/Decline to State.

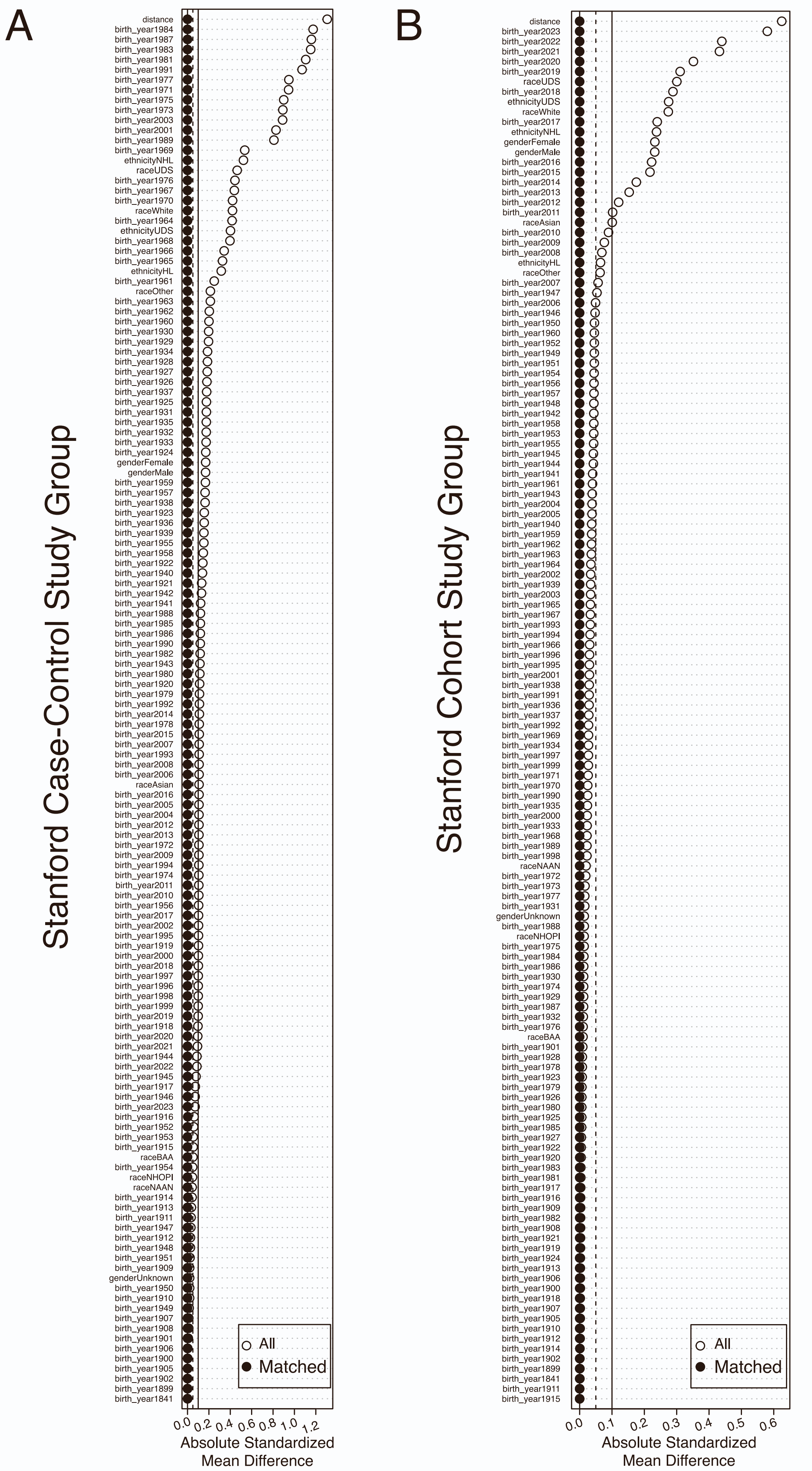

Figure S13. Stanford Matching Distances. Related to Figure 1. Absolute standardized mean differences for covariates that were used for matching in the main Stanford risk analysis, for either the case-control (A) or cohort (B) study group. HL = Hispanic/Latino, NHL = Not Hispanic/Latino, BAA = Black or African American, NAAN = Native American or Alaska Native, NHOPI = Native Hawaiian or Other Pacific Islander, UDS = Unknown/Decline to State.
